# Prussian Blue Analogue Materials for Seawater Splitting: A Review

**DOI:** 10.3390/ijms27177902

**Published:** 2026-09-04

**Authors:** Sebastian Salazar-Avalos, Víctor M. Jiménez-Arévalo, Pedro Pablo Zamora, Danny Guzman, Klaus Bieger, Álvaro Soliz, Norman Toro, Atul Sagade, Daniel Ramírez, José H. Zagal, Felipe M. Galleguillos-Madrid

**Affiliations:** 1 Centro de Desarrollo Energético Antofagasta, Universidad de Antofagasta, Antofagasta 1240000, Chile; victor.jimenez@uantof.cl; 2 Departamento de Química de los Materiales, Universidad de Santiago de Chile, Av. Libertador Bernardo O’Higgins 3363, Santiago 9170022, Chile; 3 Departamento de Química y Biología, Facultad de Ciencias Naturales, Universidad de Atacama, Av. Copayapu 485, Copiapó 1530000, Chile; pedro.zamora@uda.cl (P.P.Z.); klaus.bieger@uda.cl (K.B.); 4 Departamento de Ingeniería en Metalurgia, Universidad de Atacama, Av. Copayapu 485, Copiapó 1530000, Chile; danny.guzman@uda.cl (D.G.); alvaro.soliz@uda.cl (Á.S.); 5 Solar Energy Research Center, SERC Chile, Universidad de Antofagasta, Av. Angamos 601, Antofagasta 1270300, Chile; 6 Faculty of Engineering and Architecture, Universidad Arturo Prat, Avenida Arturo Prat 2120, Iquique 1110939, Chile; notoro@unap.cl; 7 Departamento de Ingeniería Mecánica, Universidad de Tarapacá, Arica 1100000, Chile; asagade@academicos.uta.cl; 8 Laboratorio de Electroquímica y Nanociencias (LEN), Instituto de Química, Facultad de Ciencias, Universidad de Valparaíso, Avda. Gran Bretaña 1111, Playa Ancha, Valparaíso 2360102, Chile; daniel.ramirez@uv.cl; 9 Departamento de Química de los Materiales, Facultad de Química y Biología, Universidad de Santiago de Chile, Av. Libertador Bernardo O’Higgins 3363, Santiago 9170022, Chile; jose.zagal@usach.cl

**Keywords:** Prussian Blue Analogues, electrolysis, seawater, green hydrogen, corrosion

## Abstract

Seawater electrolysis is emerging as a key alternative strategy for sustainable hydrogen production in water-scarce regions such as the Atacama Desert; however, the high concentration of chloride ions poses significant challenges related to material stability, selectivity, and corrosion resistance. In this context, Prussian Blue Analogues (PBAs) have recently gained attention as multifunctional materials capable of operating in highly saline environments such as seawater. This review provides a critical analysis of PBAs as electrocatalysts for the hydrogen evolution reaction (HER) and oxygen evolution reaction (OER) in seawater and brine systems, with a particular emphasis on nickel hexacyanoferrate (NiHCF), cobalt hexacyanoferrate (CoHCF), and copper hexacyanoferrate (CuHCF). In addition, a comparative analysis across different electrochemical applications is presented, highlighting the limited number of studies conducted under real seawater conditions. Furthermore, the limitations of current electrochemical evaluation protocols are discussed, and a framework for realistic benchmarking under saline conditions is proposed. Finally, emerging opportunities in hybrid materials and high-entropy PBAs are addressed, positioning PBAs as a promising platform for next-generation electrochemical technologies for sustainable solar hydrogen production from seawater or highly chloride-concentrated brines.

## 1. Introduction

The large-scale deployment of hydrogen technologies is widely recognized as a cornerstone for the decarbonization of modern energy systems. However, despite significant advances in water electrolysis technologies, their reliance on high-purity freshwater remains a critical bottleneck, particularly in arid regions where water scarcity and energy demand coexist. In this context, the direct utilization of seawater as an electrolyte represents an attractive yet fundamentally challenging alternative, as it introduces a complex chemical environment that severely impacts catalytic performance.

However, these electrolyser technologies are highly dependent on high-purity freshwater, as anion exchange membrane (AEM) electrolysers typically require conductivities below 20 µS cm^−1^, while proton exchange membrane (PEM) systems demand values below 1 µS cm^−1^. This requirement represents an additional constraint on their global implementation [1,2,3,4]. In this context, the direct use of saline water emerges not only as a practical alternative, but also as an opportunity to rethink electrolysis from a process-integrated perspective, in which electrolyte composition, catalytic performance, and resource recovery are intrinsically interconnected [5].

Unlike conventional electrolysis systems operating in purified electrolytes, seawater electrolysis involves a multicomponent ionic matrix dominated by high concentrations of chloride ions (Cl^−^), along with various multivalent cations such as Mg^2+^ and Ca^2+^. These species not only induce corrosion and electrode degradation, but also promote competing reactions, most notably the chlorine evolution reaction (CER), which directly competes with the oxygen evolution reaction (OER). As a result, catalytic performance in seawater cannot be evaluated solely in terms of activity, but must also consider selectivity, interfacial stability, and resistance to fouling under dynamic operating conditions. Despite extensive research efforts, achieving efficient and selective electrolysis in seawater remains an unresolved challenge.

In this context, metrics such as overpotential (η) and current density, while still relevant, are insufficient to fully describe performance unless complemented by considerations of selectivity, resistance to fouling, and long-term operational stability under realistic conditions. Seawater is an abundant and attractive alternative feedstock. Nevertheless, its direct utilization introduces substantial physicochemical challenges associated with its complex ionic composition. In particular, the high concentration of chloride ions not only accelerates anode electrode corrosion but also promotes parasitic reactions such as the CER, leading to the formation of species such as Cl_2_ and hypochlorite (ClO^−^) instead of oxygen (O_2_) [6,7,8]. These reactions directly compete with OER, thereby reducing the efficiency of the seawater electrolysis process [9,10,11]. Prussian Blue Analogues (PBAs) can be generally represented by the formula A_x_M[M’(CN)_6_]_1−γ_·zH_2_O, where A represents an interstitial alkali-metal cation, M and M’ are transition-metal centres, y accounts for the fraction of [M’(CN)_6_] vacancies, and z represents the hydration degree. The use of PBAs as cathode materials for seawater-related electrochemical processes linked to hydrogen evolution remains relatively limited; however, they have been more extensively studied as cathode materials in potassium, lithium, sodium, and other ion-based battery systems [12,13,14].

The electrocatalytic activity of PBAs for the HER in saline media highlights promising trends in terms of overpotential, stability, and kinetics. However, although overpotential values are reported for nickel hexacyanoferrate (NiHCF) and copper hexacyanoferrate (CuHCF), no direct comparison with benchmark catalysts (e.g., Pt or NiMo) is provided, making it difficult to assess the true competitiveness of these materials. Moreover, the reported values remain relatively high compared to optimized systems, suggesting that, while PBAs are functional in saline environments, they still exhibit significant limitations in terms of energy efficiency. In the case of cobalt hexacyanoferrate (CoHCF), the improved current density is attributed to enhanced electronic conductivity and favourable adsorption of intermediates; however, this interpretation lacks detailed mechanistic support. In this context, we have also examined the charge transfer mechanism associated with these systems, providing additional insight into the electrochemical behaviour of PBAs, which may be extended to the related materials discussed above [15].

The available evidence indicates that PBAs can retain electrochemical functionality over a broad range of chloride concentrations. However, differences in composition, synthesis route, defect density, and electrolyte conditions lead to substantial variations in performance, highlighting the need for standardized criteria when comparing different studies [16].

Previous reviews of PBAs have focused primarily on their use in energy storage, ion intercalation, water dissociation, oxygen evolution, and PBA-derived electrocatalysts. However, fewer studies have examined these materials specifically under the conditions imposed by seawater electrolysis, where catalytic performance is strongly affected by chloride competition, multivalent ions, salt precipitation, corrosion, and surface rebuilding during operation. From this perspective, the present review focuses on three closely related aspects: (i) electrocatalytic activity and reaction selectivity, (ii) ion transport and interactions at the material-electrolyte interface, and (iii) structural stability and evolution under chloride-rich conditions. This approach allows the analysis of synthesis, defect chemistry, hydration, surface reconstruction, and electrochemical performance as interconnected factors that determine the behaviour of PBAs in salt electrolysis systems.

This review presents a critical and integrated analysis of PBAs as electrocatalytic materials for seawater electrolysis, with a particular focus on HER and OER in saline media. The analysis centres on the relationship between structure, composition, charge transport, and electrochemical performance, also considering the limitations arising from experimental methodologies and the lack of standardized conditions among reported studies. Furthermore, the main PBA systems are compared with other reference catalysts, and emerging strategies, such as defect engineering, hybrid materials, multimetal systems, and high-entropy PBAs, are discussed. Thus, the integration of experimental evidence and mechanistic principles allows for the identification of the main current limitations and the establishment of design criteria to guide the development of more active, selective, and stable PBAs for operation in seawater and other media with high chloride concentrations.

## 2. Electrochemical Behaviour of PBAs in Contact with Seawater

From the anodic perspective, PBAs may offer several advantages over conventional materials used in seawater electrolysis. Their coordination environment can stabilize reaction intermediates, while their structural flexibility enables dynamic behaviour under high-salinity operating conditions [17]. Furthermore, the presence of cyanide ligands introduces an electronic environment distinct from that of oxides or alloys, which can influence the adsorption energetics of key intermediates such as *H, *OH, *O, and *OOH, thereby modulating the competition between OER and the CER [18,19,20].

At the cathode, the HER proceeds primarily via water reduction in neutral or alkaline saline media, as described in Equation (1).

(1) 2 H_2_O + 2 e^−^ → H_2_ + 2 OH^−^

However, during the cathodic process, the HER may compete with the oxygen reduction reaction (ORR) due to the presence of dissolved oxygen, particularly under open or poorly controlled conditions, as described in Equation (2).

(2) O_2_ + 2 H_2_O + 4 e^−^ → 4 OH^−^

Although ORR is thermodynamically more favourable, its slower kinetics typically limit its contribution under operational conditions. Equation (3) represents the alternative route (with a 2 e^−^ mechanism), which is energetically undesirable, as described in Equation (3).

(3) O_2_ + H_2_O + 2 e^−^ → HO_2_^−^ + OH^−^

More critically, HER in seawater is strongly influenced by local pH variations and ion accumulation at the electrode surface, which can lead to the precipitation of insoluble hydroxides such as Mg(OH)_2_ and Ca(OH)_2_. These deposits can block active sites, reduce effective surface area, and increase mass transport resistance, ultimately decreasing hydrogen production efficiency. At the anode, the situation becomes significantly more complex due to the direct competition between the OER in Equation (4) and the CER in Equation (5).

(4) 4 OH^−^ → O_2_ + 2 H_2_O + 4 e^−^

(5) 2 Cl^−^ → Cl_2_ + 2 e^−^

(6) Cl_2_ + 2 OH^−^ → Cl^−^ + ClO^−^ + H_2_O

(7) Cl^−^ + 2OH^−^ → ClO^−^ + H_2_O + 2e^−^

While the OER is the desired pathway for oxygen production, the CER becomes increasingly favourable in chloride-rich environments, both thermodynamically and kinetically. Under alkaline conditions, the oxidation of Cl^−^ can lead to the formation of Cl_2_, which can subsequently react to produce ClO^−^ (see Equations (6) and (7)), effectively lowering the apparent potential required for CER and intensifying its competition with OER [21,22]. Otherwise, the potential of the CER is significantly reduced due to the conversion of Cl_2_ to ClO^−^, allowing CER to occur at potentials very close to those of the OER.

### Scope and Limitations of Density Functional Theory (DFT) for Understanding HER on PBAs

Density Functional Theory (DFT) provides useful insight into the local chemistry involved in the HER on PBAs, particularly regarding water activation, electronic redistribution, and the stabilization of reaction intermediates. However, these results must be interpreted within the limits of the computational model. A molecular reaction pathway describes the energetic behaviour of a selected atomic environment, whereas the experimentally measured electrochemical response arises from a much more complex electrode–electrolyte interface. This distinction becomes particularly important in saline media, where charge transport, hydration, structural defects, chloride interactions, and surface reconstruction may simultaneously affect the reaction.

CuHCF provides a useful model for illustrating this distinction because its Fe–CN–Cu framework combines redox-active metal centres with a coordination environment capable of accommodating local electronic changes [23]. Following a computational approach similar to that reported by Bergendahl et al. [24], calculations were performed at the ωB97X-D/6-311G(d,p) level of theory. The ωB97X-D functional provides an improved description of long-range exchange and dispersion interactions, which is relevant for describing charge redistribution and weak interactions within coordination systems [25,26].

**Figure 1:**
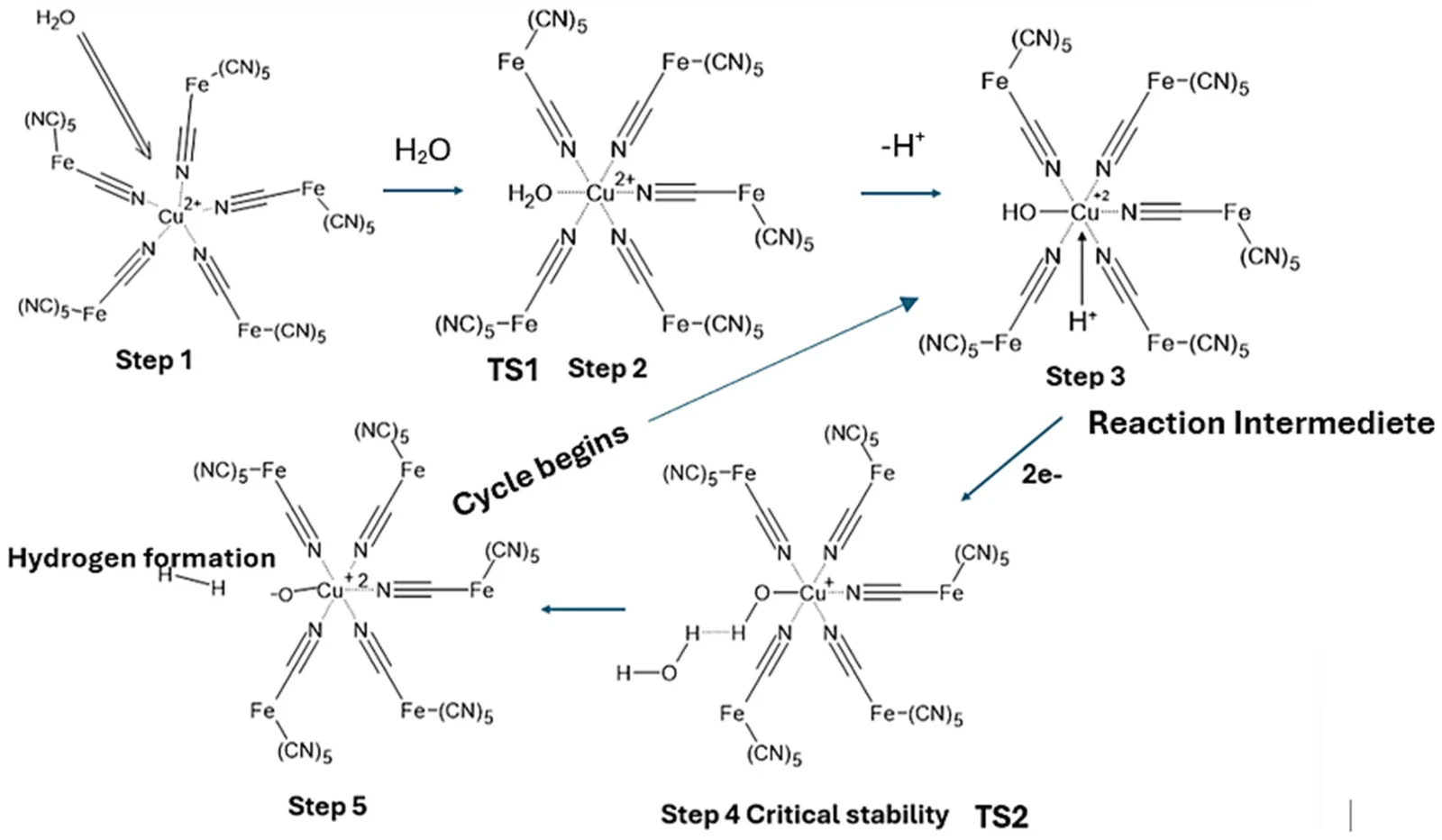
Proposed local molecular pathway for water activation and HER-related transformations in a CuHCF coordination environment. The pathway represents a simplified description of local reaction chemistry and should not be interpreted as a universal HER mechanism for PBAs.

The reaction pathway considered for CuHCF begins with the interaction of H_2_O with the Cu coordination environment (Figure 1). The first transition state (TS1) presents an activation barrier of approximately 12 kcal mol^−1^ and is associated with water activation and local reorganization around the Cu site. This suggests that the initial interaction with water represents an energetically relevant step within the molecular pathway. However, this barrier should not be interpreted as the direct origin of the experimental HER overpotential, since it only describes the intrinsic energetic cost of a local molecular transformation.

**Figure 2:**
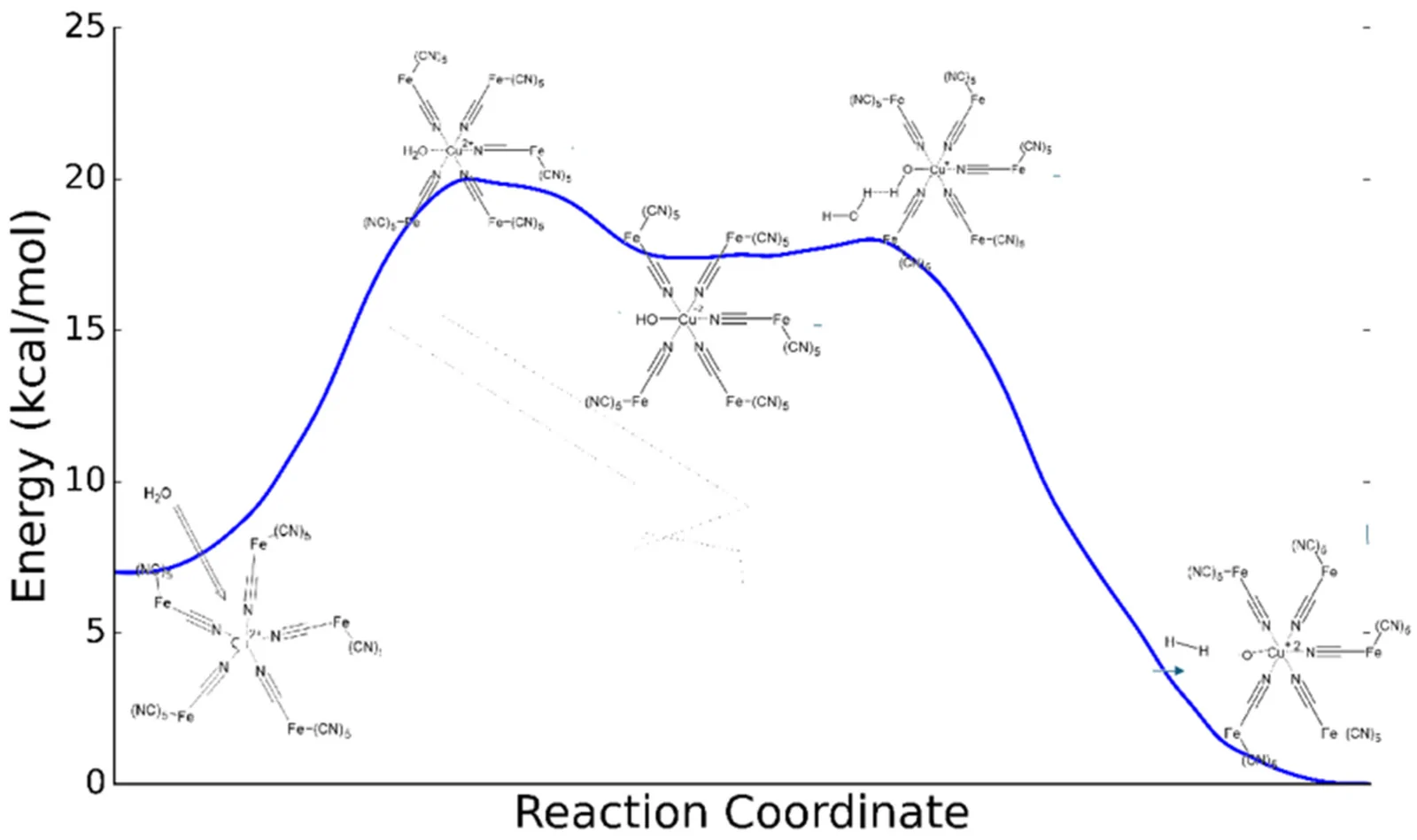
Intrinsic reaction coordinate (IRC) energy profile for the investigated HER-related pathway in the CuHCF molecular model. The energy barriers describe transformations along the selected molecular reaction coordinate and should not be interpreted as experimental electrochemical overpotentials.

After formation of the Cu–OH-containing intermediate, the pathway proceeds toward a second transition state (TS2), with a substantially lower calculated barrier of approximately 1–2 kcal mol^−1^ (Figure 2). In this case, the difference between TS1 and TS2 is more informative than the absolute barrier values. Once water activation has occurred and the intermediate has formed, the subsequent local transformation becomes considerably more accessible. The calculation therefore suggests that water activation requires greater local structural and electronic rearrangement than the later hydrogen-forming steps [27].

The electronic configuration of Cu provides a reasonable interpretation of this behaviour. Cu(II), with a d^9^ configuration, can accommodate distorted coordination geometries, whereas reduction toward Cu(I), with a d^10^ configuration, favours lower coordination numbers [28]. This change can modify the interaction between the metal centre and the adsorbed intermediate and promote electronic redistribution. Nevertheless, an energetically accessible transformation does not necessarily identify the rate-determining step of the operating electrode. Charge must still reach the active site through the cyanide-bridged framework, and this process can be affected by crystallinity, vacancies, hydration, and electronic connectivity [27,28,29,30,31].

The recent study by Salazar-Avalos et al. [31] on BiHCF provides a useful comparison because it examines a chemically different metal centre within a PBA framework. In that study, a simplified BiHCF coordination model showed relatively low barriers for water coordination (~6 kcal mol^−1^), deprotonation (~2 kcal mol^−1^), and a subsequent hydrogen-forming step (~1 kcal mol^−1^). These results indicate that the local Bi site can, in principle, accommodate elementary transformations associated with water activation and hydrogen formation.

The experimental response of BiHCF, however, shows why these molecular barriers should not be considered in isolation. HER-related Tafel slopes of approximately 153–169 mV dec^−1^ were obtained in artificial NaCl electrolytes, and the electrochemical response became increasingly limited under hypersaline conditions [31]. Post-electrochemical characterization also indicated the formation of a BiOCl-rich surface region. Thus, although the calculated pathway suggests that the pristine Bi coordination environment does not impose a large intrinsic barrier to the investigated elementary steps, the real electrode still exhibits substantial polarization. Rather than representing a disagreement between theory and experiment, this difference indicates that molecular energetics account for only part of the overall electrochemical response.

The comparison between CuHCF and BiHCF is therefore more useful from a mechanistic perspective than from a numerical comparison of their activation barriers. In both cases, DFT identifies energetically feasible local transformations. However, the BiHCF results demonstrate experimentally that favourable local chemistry does not necessarily translate into a low macroscopic overpotential. Charge transport through the electrode, interfacial resistance, electrolyte composition, water activity, and surface reconstruction can become equally or even more important under operating conditions.

This distinction is particularly relevant when comparing calculated activation barriers with electrochemical overpotential. An IRC calculation describes the energetic pathway between molecular configurations, whereas overpotential represents the additional electrode potential required to sustain an electrochemical reaction at a given rate. Consequently, a barrier expressed in kcal mol^−1^ cannot be directly converted into an HER overpotential. Establishing such a relationship would require an explicit treatment of the electrode potential and a more complete description of the electrochemical interface, including potential-dependent solvation and interfacial charge.

Chloride introduces an additional limitation to simplified models. Cl^−^ is not explicitly included in the CuHCF pathway and, therefore, its influence cannot be assumed to be negligible simply because it does not participate in the calculated reaction coordinate. BiHCF provides a clear example: chloride is not required to explain the intrinsic feasibility of water activation at the Bi site, yet experimentally it promotes the formation of a BiOCl-rich interphase that modifies the electrode response [31]. This highlights why DFT and experimental characterization should be considered complementary rather than interchangeable approaches [29,30].

The same reasoning limits the generalization of the CuHCF pathway to other PBAs. Although the cyanide-bridged framework is a common structural feature, the local reaction chemistry remains strongly dependent on the metal centre. Cu, Bi, Ni, Co, and Fe differ in electronic configuration, redox flexibility, coordination preferences, and their interactions with reaction intermediates. Vacancies and hydration further modify these properties. The CuHCF pathway should therefore be regarded as an example of how a PBA metal centre may participate in HER-related chemistry rather than as evidence of a universal mechanism for the entire PBA family [32,33].

This consideration is also relevant when discussing overpotential. As described by Raveendran et al. [34], the experimentally observed overpotential reflects several contributions associated with electron transfer, adsorption and desorption of intermediates, and changes at the electrode surface. In chloride-rich systems, these effects become particularly important because the interface may evolve during polarization. Accordingly, molecular activation barriers can help identify intrinsically demanding reaction steps, but they cannot by themselves account for the potential required to sustain the reaction under practical operating conditions.

From this perspective, DFT is particularly valuable for the rational study of PBAs when it is used to test mechanistic hypotheses rather than to predict electrochemical performance in isolation. It can help identify plausible elementary transformations, examine how the metal centre affects intermediate stabilization, and evaluate the influence of local coordination. Experimental measurements are then needed to determine how these molecular features are expressed at the actual electrode interface.

For seawater electrolysis, this combined approach remains especially important because the interface is dynamic and chemically complex. Multicomponent ion interactions, local pH gradients, bubble formation, surface heterogeneity, and competitive adsorption can modify apparent kinetic parameters such as exchange current density and Tafel slope. Consequently, comparisons between PBA electrocatalysts should be made cautiously when experimental conditions are not standardized [34,35,36,37,38]. Future computational studies incorporating structural vacancies, explicit hydration, chloride interactions, and potential-dependent interfaces should help narrow the current gap between local molecular energetics and the electrochemical behaviour of PBAs under realistic saline conditions.

## 3. General Characteristics of PBAs

**Figure 3:**
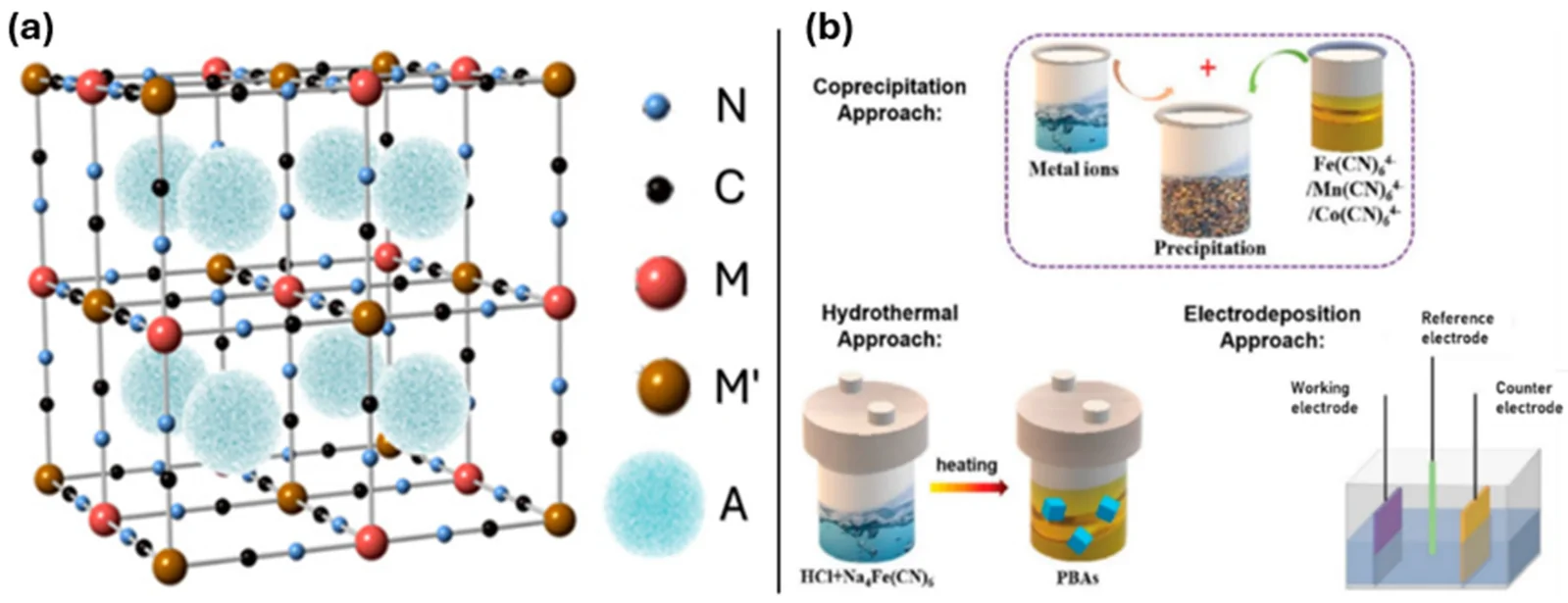
(**a**) Unit cell of PBA. Based on Galleguillos et al. (2020) [59]; (**b**) Schematic diagram of the PBA synthesis process by coprecipitation, hydrothermal and electrodeposition methods. Based on Zhao et al. (2020) [60].

PBAs are a family of coordination compounds [24] derived from the original Prussian Blue, with the simplified idealized representation M[M’(CN)_6_]·H_2_O, where M and M’ are transition metal cations (Figure 3a). These materials exhibit an inverted perovskite-type face-centred cubic crystal lattice, in which metal ions are connected by cyanide groups, forming a highly porous and well-ordered three-dimensional framework [39,40,41,42,43]. This type of crystal lattice gives PBAs unique properties for applications in energy storage, batteries, sensors and, more recently, in electrocatalysts for saline water splitting [27,43,44,45,46].

The coordination environment in PBAs provides a unique electronic structure, distinct from that of conventional oxides or alloys. The presence of strong-field cyanide ligands modifies the electronic configuration of the metal centres, thereby influencing the adsorption energetics of key reaction intermediates. In addition, structural defects, such as [M’(CN)_6_] vacancies and coordinated water molecules, introduce local distortions that may act as active sites, although they can also compromise structural stability under electrochemical polarization. Therefore, understanding the interplay between the pristine PBA structure and its dynamically evolving surface is essential to rationalize their electrocatalytic behaviour in complex electrolytes such as seawater [47].

One of the most important characteristics of PBAs is the diversity of their analogues, which enables the incorporation of a wide range of transition metals (such as Fe, Co, Ni, Mn, Cu, and Zn) within the same crystalline framework. This multimetallic nature allows fine-tuning of the electronic structure and redox potentials through metal substitution, providing an effective strategy to modulate catalytic activity [31,48,49,50]. In particular, the choice of metal centres directly governs the electronic structure of the active site, thereby modulating the adsorption strength, stability, and reactivity of key intermediates formed during both cathodic and anodic processes. This effect is intrinsically linked to the d-electron configuration of the metal, which determines the energy, occupancy, and spatial orientation of the frontier orbitals involved in bonding with adsorbed species [51]. In the case of CuHCF, the Cu(II) centre (d^9^) exhibits partially filled d orbitals that can effectively interact with the lone pairs of adsorbed species such as H_2_O and OH^−^, facilitating the initial polarization and activation of the H–O bond associated with the TS1 step. However, upon reduction to Cu(I) (d^10^), the electronic configuration becomes fully filled, leading to a decrease in directional bonding and a preference for lower environments with lower coordination numbers. This transition reduces the ability of the metal centre to stabilize adsorbed intermediates within the original coordination environment, favouring electronic redistribution toward the adsorbed OH species and facilitating the subsequent steps involved in hydrogen evolution.

Importantly, this behaviour is not solely a consequence of local coordination chemistry but also reflects how the electronic structure of the metal centre couples with the surrounding PBA lattice. Since charge must be delivered to the active site through the cyanide-bridged network, the accessibility of these d orbitals under operating conditions depends on the ability of the framework to sustain electron flow. As a result, the adsorption strength of intermediates is not static, but dynamically modulated by the local electronic population at the metal site, particularly during the transition from the activation-controlled regime (TS1) to the charge-driven regime (TS2).

When extended to other PBAs, differences in metal identity (e.g., Ni, Co, Fe) lead to variations in d-band filling, redox flexibility, and preferred coordination geometries, which in turn shift the balance between adsorption strength and intermediate stability. The common cyanide-bridged framework allows general structure–property principles to be compared; however, a common HER pathway cannot be assumed. Differences in metal identity, redox flexibility, coordination environment, hydration, and vacancy chemistry may generate material-specific reaction pathways and intermediate energetics [52].

Studies on PBAs highlight advances in (i) synthesis, (ii) structural control, and (iii) performance in adsorption and ion intercalation applications [53]. In the case of Su et al. [54], a significant increase in surface area is reported, which enhances kinetics; however, the underlying mechanisms responsible for these improvements are not fully explored, particularly in relation to defect distribution and active site accessibility. In contrast, the study by Ullah et al. [55] provides relevant insights into ion selectivity and diffusion coefficients in saline media; however, the analysis does not connect these findings to broader implications for complex electrochemical systems, where competition between ionic species and material stability are critical factors.

This apparent relationship between crystallinity and catalytic performance can be rationalized by considering the structural and electronic nature of PBAs. Highly crystalline materials exhibit a well-ordered Fe–CN–M framework, which not only enhances long-range electronic connectivity by facilitating more coherent electron hopping pathways between metal centres, but also provides well-defined channels and uniformly accessible metal sites. In such systems, the cyanide bridge acts as an efficient electronic mediator, allowing intervalence charge transfer between Fe^2+^/Fe^3+^ and M^2+^/M^+^ redox couples, which is beneficial for sustaining charge propagation across the lattice [46]. As a result, increased crystallinity does not necessarily reduce the number of active sites but rather can improve their accessibility and electronic coupling within the framework.

At the same time, the presence of structural defects, such as vacancies or local distortions, can further modulate catalytic behaviour by altering the coordination environment of metal centres and tuning adsorption energies. However, excessive disorder may disrupt electronic communication between sites and hinder efficient charge transport. Therefore, catalytic performance in PBAs is not governed by crystallinity alone, but by a balance between structural order, which ensures efficient charge propagation and accessibility, and controlled defect density, which can enhance local reactivity without compromising lattice connectivity.

These studies demonstrate a clear trend in the development of PBAs, where control over synthesis emerges as a key parameter for tailoring the material’s structure and size, and consequently its functional performance. Both the studies by Su and Ullah et al. [54,55] optimize morphology through controlled co-precipitation, adjusting the pH to promote nucleation and thereby obtaining highly crystalline and homogeneous structures, which enhance electrochemical stability. In parallel, Zeng et al. [23] demonstrate that these structural characteristics crystalline order, active site density, and open channels directly influence electrochemical processes, which are governed by diffusion and selectivity. This convergence suggests that a rational synthesis design enables the optimization of PBAs as multifunctional materials (e.g., selective adsorption, energy storage, or electrochemical desalination), with high adaptability for applications in saline media, positioning them as promising candidates for electrolysis and selective ion separation processes.

The pseudocapacitive behaviour reported by various researchers, associated with reversible redox processes at the surface and within the structure of the material, not only facilitates the adsorption and exchange of reactive species such as H^+^ and OH^−^, but also promotes faster charge-transfer kinetics. There is limited information regarding the potential influence of PBAs on mass transport processes. These characteristics enable materials such as NiHCF and CoHCF to maintain high structural stability in aggressive media, as well as efficient ionic transport, both of which are essential conditions for electrolysis applications in saline solutions [56,57,58].

The black and blue spheres represent the bridging ligand –C≡N–, which connects the metal centres. The red sphere corresponds to the transition metal M, in typical coordination with C. The orange sphere corresponds to the second transition metal M’, in coordination with N. The diffuse light blue sphere corresponds to the interstitial space for the accommodation of the cation in the process of ionic intercalation [59,61]. As highlighted by Nordstrand et al. [46], PBAs present well-defined diffusion channels for ions, being a distinct advantage over traditional metal oxides, making them especially promising in aggressive environments such as seawater, where the combined selectivity, activity, and stability of the material are key.

## 4. PBA Synthesis

The synthesis of PBAs plays a crucial role in determining their structural, electronic, and electrochemical properties. Unlike conventional catalytic systems, where composition is often the primary design variable, in PBAs the synthetic route directly governs key parameters such as crystallinity, defect density, particle size, hydration level, and the distribution of active metal centres. In general, PBA synthesis can be categorized into solution-based methods (Figure 3b), such as coprecipitation and hydrothermal synthesis, and electrochemical or solid-state approaches, including electrodeposition and mechanochemical (ball milling) routes. Each method provides a distinct pathway to control nucleation and crystal growth, leading to materials with markedly different structural characteristics. For instance, rapid nucleation processes tend to produce highly defective and hydrated frameworks, whereas slower growth conditions favour increased crystallinity and reduced vacancy concentration. These differences are not merely structural, but have direct implications on electrochemical behaviour, particularly in terms of electronic conductivity, ion diffusion, and stability under polarization [42,62,63,64,65,66].

Importantly, the presence of structural defects, such as [M’(CN)_6_] vacancies (see Figure 3), interstitial water molecules, and coordination disorder, emerges as a critical factor linking synthesis to performance. While defects can enhance ionic transport and increase the number of accessible active sites, they may also weaken the structural integrity of the framework and accelerate degradation in aggressive environments. This dual role highlights the need for controlled defect engineering, in which the density and distribution of vacancies are optimized to balance catalytic activity and long-term stability. In this context, the choice of synthesis method should not be regarded merely as a preparation step, but as a fundamental tool for tuning the physicochemical properties of PBAs towards specific electrochemical applications [45,67].

### 4.1. Co-Precipitation

Co-precipitation is the most widely used method for the synthesis of PBAs due to its simplicity, scalability, and ease of operation. The process typically involves the mixing of a transition metal salt (e.g., Ni^2+^, Co^2+^, Cu^2+^) with a hexacyanometallate precursor, such as K_3_[Fe(CN)_6_] or K_4_[Fe(CN)_6_], in aqueous solution, leading to the spontaneous formation of a colloidal precipitate. Despite its apparent simplicity, the coprecipitation route is highly sensitive to synthesis parameters, including (i) precursor concentration, (ii) mixing rate and pH, and (iii) temperature, all of which critically determine nucleation kinetics and subsequent crystal growth. This method often produces PBAs with significant morphological defects, substantial amounts of interstitial water, and heterogeneous particle size distributions [68,69,70,71,72].

Camacho et al. [63] obtained PBA through coprecipitation at 60 °C, obtaining grain sizes of 50 nm that aggregate into 10 µm particles. The rapid nucleation of this route favours irregular aggregates compared to well-defined cubes of other routes. From Thermogravimetric Analysis coupled with Mass Spectrometry (TGA-MS) and Powder X-ray diffraction (PXRD), they propose for the coprecipitated sample a composition close to Na_1.4_Fe_2_(CN)_6_ 1.6·H_2_O and point out [Fe(CN)_6_] vacancies a framework composed of mixed-valence hexacyanoferrate units ([Fe(CN)_6_]^3−^/[Fe(CN)_6_]^4−^) and a higher Fe^3+^ fraction (57%), consistent with more defective and hydrated material than its hydrothermal analogue [73,74].

In contrast, Zhang et al. [75] introduced a hydrodynamic control method, achieving a reduction in vacancy and interstitial water formation, resulting in a more stable crystal lattice and significant improvements in the material’s capacity and cyclability. Therefore, rigorous control of mixing conditions and nucleation kinetics allows for direct modulation of defect density and interstitial water content, critical parameters that determine the electrochemical performance of PBAs.

### 4.2. Hydrothermal Synthesis

Hydrothermal synthesis represents a powerful approach for producing PBAs with enhanced crystallinity, controlled morphology, and reduced defect density compared to coprecipitation methods. This technique involves the treatment of precursor solutions in sealed autoclaves at elevated temperatures and pressures, typically promoting slower nucleation and more uniform crystal growth. As a result, hydrothermally synthesized PBAs generally exhibit improved structural order, lower concentrations of [M’(CN)_6_] vacancies, and reduced interstitial water content. These features contribute to enhanced structural stability and more defined ion diffusion pathways, which are particularly advantageous for applications requiring long-term electrochemical operation [76,77,78]. From a physicochemical perspective, the controlled growth environment in hydrothermal synthesis enables fine tuning of particle size, morphology, and phase composition. Importantly, variations in temperature, pH, and reaction time can induce partial hydrolysis of the PBA framework, leading to the in situ formation of metal oxide or hydroxide phases within or on the surface of the material. This phenomenon is particularly relevant for electrocatalysis, as these secondary phases, such as FeOx, Ni(OH)_2_, or mixed (oxy)hydroxides, are widely recognized as the true active species for the OER. From an electrochemical perspective, hydrothermal synthesis is closely linked to the balance between structural integrity and catalytic activation. Highly crystalline PBAs tend to exhibit greater stability and reduced degradation in aggressive electrolytes; however, their lower defect density may limit the number of accessible active sites and slow down ion transport. Conversely, the controlled introduction of secondary phases or surface defects can enhance catalytic activity, particularly for anodic reactions, but may compromise long-term durability.

Yang et al. [79] prepared Fe_3_[Fe(CN)_6_]_2_ nanocrystals using a hydrothermal method at a low temperature of 80 °C for 12 h, starting from K_3_[Fe(CN)_6_] in an acidic medium. The researchers propose a mechanism involving the partial dissociation of [Fe(CN)_6_]^3−^ followed by the protonation of the released CN^−^ species to form HCN, which can undergo hydrolysis or further decomposition under acidic hydrothermal conditions, generating carbon-containing species such as CO. The study suggests that this species acts as a reducing agent of Fe^3+^ to Fe^2+^, favouring uniform nucleation. Yang postulates that the size of the resulting grain depends strongly on the acidity and concentration of the precursor; therefore, to obtain a greater number of nuclei and smaller crystals, the synthesis should be carried out with higher acidity and concentration of K_3_[Fe(CN)_6_]. With respect to crystallinity, a cubic structure was obtained (lattice parameter a = 10.19 Å), with a clean surface associated with better electron transfer kinetics and superior electrochemical performance.

Zhang et al. [80] used a hydrothermal method to synthesize PBA, controlling the temperature from 100 to 260 °C for a period of 2 to 12 h. They reported that, under these conditions, the M–CN–Fe structure undergoes hydrolysis, leading to the release of CN^−^ species and the progressive formation of iron oxide phases Fe(OH)_3_ → Fe_2_O_3_ → Fe_3_O_4_ → Fe_0.942_O, depending on the temperature and reaction time. This decomposition involves not only a hydrolysis process but also the partial reduction of Fe^3+^ to Fe^2+^, where the necessary electrons likely originate from the oxidation of cyanide ligands or their decomposition products under hydrothermal conditions. Consequently, the formation of PBA via a hydrothermal process at temperatures close to 260 °C results in a structure with FeOx nanoparticles embedded within the material’s porosity.

The researchers also correlated other variables such as pH, particle size, and crystalline phase with the OER overpotential at 10 mA cm^−2^. The results obtained demonstrate that greater porosity and a specific FeOx phase are associated with improved kinetics. It is important to mention that iron oxides are known for their electrocatalytic activity; therefore, their greater presence improves performance in combination with the intrinsic activity of FeOx and the PBA structure. The findings reported by Zhang highlight hydrothermal synthesis as a viable strategy for tuning OER activity by controlling texture, phase composition, and surface chemistry [81,82].

In the case of Yang et al. [79], the hydrothermal process at low temperature (80 °C) is oriented towards preserving the PBA structure, promoting controlled nucleation through redox mechanisms induced by the partial decomposition of the cyanide ligand. This approach enables the formation of highly crystalline materials with clean surfaces and enhanced electron transfer kinetics. The strategy focuses on the fine control of particle size and structural homogeneity, where parameters such as acidity and precursor concentration directly govern nucleation. However, the study does not explore in depth the possible formation of secondary active phases, nor how these structures behave under electrochemical operation in aggressive environments.

In contrast, Zhang et al. [80] adopt a more thermally aggressive approach (up to 260 °C), in which the PBA structure undergoes partial transformations induced by hydrolysis, leading to the in situ formation of iron oxide (FeO_x_) phases. Unlike Yang’s work, this partial degradation of the framework is not considered a limitation but rather a functional advantage, as these phases are widely recognized as the true active species for the OER. This approach represents a transition from “pristine” PBAs to hybrid PBA–oxide materials, where electrocatalytic activity is dominated by the secondary phase rather than the original framework. Taken together, these studies demonstrate that the hydrothermal synthesis of PBAs offers a greater degree of control over nucleation, crystallinity, and phase evolution compared to the coprecipitation method, allowing for fine-tuning of both the material’s structure and functionality. Yang shows that at low temperatures and in an acidic medium, nanocrystals with a high degree of crystallinity can be obtained, where the precursor concentration governs particle size and structural uniformity. Zhang, in turn, demonstrates that at higher temperatures, partial decomposition of the M–CN–Fe network occurs, generating secondary FeO_x_ phases that enhance the material’s catalytic properties. In this context, a clear relationship exists between temperature, pH, and time conditions and the degree of crystallinity, and consequently, the electrochemical performance offered by the hydrothermal method, which yields more ordered structures with fewer defects.

### 4.3. Electrodeposition

Electrodeposition offers a direct and versatile strategy for the fabrication of PBA-based electrodes with controlled thickness, morphology, and strong interfacial adhesion to conductive substrates. Unlike bulk synthesis methods, electrodeposition enables the in situ formation of PBA films on current collectors such as glassy carbon, fluorine-doped tin oxide (FTO), or metallic foams, providing intimate electrical contact and minimizing interfacial resistance. This characteristic is particularly advantageous for electrochemical systems operating in saline media, where efficient charge transfer and mechanical stability at the electrode–electrolyte interface are critical under continuous operation.

A key advantage of electrodeposition lies in its ability to tailor the nucleation and growth of PBAs under electrochemical control. Parameters such as applied potential, current density, deposition time, and electrolyte composition directly influence film thickness, crystallinity, and defect distribution. In chloride-containing electrolytes, these factors also determine how the material interacts with aggressive ionic species, including Cl^−^, Mg^2+^, and Ca^2+^. Thin and conformal PBA coatings can enhance ion accessibility and facilitate mass transport, while maintaining sufficient electronic connectivity for efficient HER and OER processes. At the same time, controlled film growth can mitigate surface degradation, delamination, and poisoning effects commonly observed in seawater electrolysis.

From a functional perspective, electrodeposited PBAs are particularly well suited for integrated electrochemical systems combining electrocatalysis and ion separation. Their thin-film architecture enables rapid ion exchange and surface redox activity, which can contribute to both hydrogen evolution at the cathode and selective ion capture or transformation at the electrode–electrolyte interface. Moreover, the direct integration of PBAs onto conductive supports allows for improved operational stability under conditions where gas evolution, local pH gradients, and salt precipitation (e.g., Mg(OH)_2_, Ca(OH)_2_) can significantly impact performance. Consequently, electrodeposition emerges as a key technique for bridging material design and device implementation, enabling the development of robust and multifunctional electrodes for seawater electrolysis [83,84].

The research by Itaya et al. [85] demonstrates a robust method for obtaining PBA films with high adhesion. This was achieved through galvanostatic cathodic polarization, using an equimolar mixture of FeCl_3_ (20 mM) and K_3_[Fe(CN)_6_] (20 mM) in 0.01 M HCl. Ideally, an oxygen-free atmosphere is required to prevent Fe^2+^ reoxidation and ensure stable growth and adhesion in the film.

Similarly, the research conducted by Steen et al. [86] on the cathodic electrodeposition of Ni-based PBA films, using polarization cycles between and 0 mV vs. SCE (saturated calomel electrode), in a solution containing 2 mM NiSO_4_, 2 mM K_3_[Fe(CN)_6_], and 250 mM Na_2_SO_4_, has yielded important results regarding structural characteristics. Although the study focuses on ion exchange, it also shares information relevant to electrocatalysis, such as the lattice parameter, which increases when transitioning from K^+^ to Cs^+^, indicating the formation of a more open PBA structure. Extended X-ray Absorption Fine Structure (EXAFS) analysis revealed a Ni coordination number of 4.4–5.1, consistent with the presence of [Fe(CN)_6_] vacancies (0.6 < and < 1.1) and local structural disorder. These characteristics regarding vacancies and network expansion can improve electrolyte accessibility, increase the number of exposed active sites, and facilitate charge transfer at the electrode/electrolyte interface. However, they can also negatively affect structural stability and reversibility in redox processes.

Electrodeposition offers an effective and useful strategy for obtaining PBA films directly integrated into an electrode, where electrochemical control is fundamental for adjusting both the structure and functionality of the material. Itaya demonstrates the formation of highly adherent films with well-defined channels that promote ion transport. Steen, for their part, demonstrate the modulation of the electrolyte potential and composition to synthesize an expanded PBA crystalline network, increasing the accessibility and density of active sites. In this context, electrodeposition is a viable alternative for designing PBAs with interfacial properties, allowing for precise adjustment of defectology, ion accessibility, and charge transfer, which is relevant for electrochemical applications [85,86].

### 4.4. Ball Milling

Mechanochemical synthesis via ball milling represents an emerging solvent-free approach for the preparation of PBAs, offering unique control over structural order, defect chemistry, and compositional complexity. In this method, chemical reactions are driven by mechanical energy through repeated collisions between precursor particles, enabling bond breaking and reformation in the solid state. Compared to conventional solution-based routes, ball milling can suppress excessive incorporation of interstitial water and reduce uncontrolled vacancy formation, leading to materials with improved structural homogeneity and enhanced stability under electrochemical conditions.

From a materials design perspective, ball milling enables the synthesis of PBAs with tunable multimetallic compositions, facilitating the incorporation of multiple transition metals within a single framework. This feature opens the possibility of designing compositionally complex or high-entropy PBA systems, where synergistic interactions between different metal centres can modulate electronic structure, redox activity, and adsorption properties. In saline electrochemical environments, such compositional flexibility may be particularly advantageous for balancing catalytic activity and corrosion resistance, as different metal species can contribute to stabilizing the framework or selectively interacting with ionic species such as Cl^−^ or OH^−^.

In addition to compositional control, mechanochemical routes allow the direct integration of conductive phases, such as carbon nanotubes, graphene, or other carbonaceous materials, during synthesis, significantly improving electronic conductivity and charge transfer kinetics. This is especially relevant for PBAs, whose intrinsically low conductivity limits their performance in electrocatalytic applications. However, as with other synthesis methods, trade-offs remain; while ball-milled PBAs can exhibit enhanced structural stability and improved conductivity when combined with conductive additives, the degree of crystallinity and long-range order may be reduced depending on milling conditions. Therefore, optimizing milling parameters, such as time, energy input, and precursor ratios, is essential to achieve a balance between structural integrity, electronic transport, and electrochemical activity, particularly in demanding environments such as seawater electrolysis [44,66,87,88,89].

Ball milling offers a robust strategy for controlling the composition and defects of PBAs. As a solvent-free mechanochemical method, it significantly reduces defects and eliminates interstitial water compared to other aqueous-phase methods. This concept was demonstrated by Cheng et al. [88] through their synthesis of PBAs with a low percentage of Fe(CN)_6_ vacancies and no interstitial water, improving structural stability and electrochemical performance. Xu et al. [89], for their part, highlight the method’s versatility for designing multimetallic and hybrid materials, making it a strategy for significantly improving electron transport and efficiency in electrochemical applications.

Taken together, these synthesis strategies highlight that the role of PBAs in saline electrochemical systems is not solely determined by their composition, but by the interplay between crystallinity, defect distribution, and phase evolution. Importantly, these parameters directly influence not only catalytic activity but also ion transport, surface reconstruction, and stability under chloride-rich environments. Therefore, synthesis should be understood as a key design tool for tuning multifunctional behaviour, rather than merely a route for material preparation.

## 5. Morphological and Elemental Characterization of PBAs

In line with the previous section on synthesis methods, it is essential to recognize the structural and morphological variability that can result from the choice of synthetic route. Even when using the same compounds or precursors, the choice of synthesis method substantially modifies the nucleation and crystal growth process, leading to materials with different structural, morphological, and defect characteristics. Parameters such as temperature, pH, reaction time, precursor and solvent concentrations, addition rate, use of complexing agents, and electrochemical conditions control the degree of crystallinity, particle size, morphological distribution, and defect density. In this sense, the structural, morphological, and elemental diversity of PBAs plays a fundamental role in their electrochemical response, manifesting itself at the micro- and nanoscale [1,46,90,91,92].

Detailed characterization of the material becomes an indispensable preliminary step before evaluating its electrochemical performance, using techniques such as scanning electron microscopy (SEM), X-ray diffraction (XRD), X-ray photoelectron spectroscopy (XPS), Fourier Transform Infrared Spectroscopy (FTIR), and Raman spectroscopy (Raman), among others. This allows for establishing direct relationships between the synthesis method and key parameters such as the presence of [M’(CN)_6_] vacancies, interstitial water content, and the accessibility of active sites.

The use of characterization techniques such as SEM allows us to obtain information on particle size uniformity, edge and face definition, degree of aggregation, and compaction between particles. From a functional perspective, it also provides morphological information relevant to electrolyte penetration and gas bubble evacuation, parameters that are relevant in PBA-type materials. These parameters enable the evaluation of the final architecture’s quality and, consequently, the accessibility of ion channels, the effective distribution of active sites, and the continuity of transport pathways, which can help rationalize their electrochemical behaviour under operational conditions.

In SEM micrographs, the presence of particles with a narrow size distribution and well-faceted geometries is indicative of controlled crystal growth, where the nucleation and growth process occurs in an orderly and reproducible manner. Conversely, a broad particle size distribution with poorly defined edges and dense, porous aggregates reflects an uncontrolled nucleation process, lacking clear organization and uniform connectivity. This results in a heterogeneous distribution of active sites and inefficient ion/electron transport pathways.

**Figure 4:**
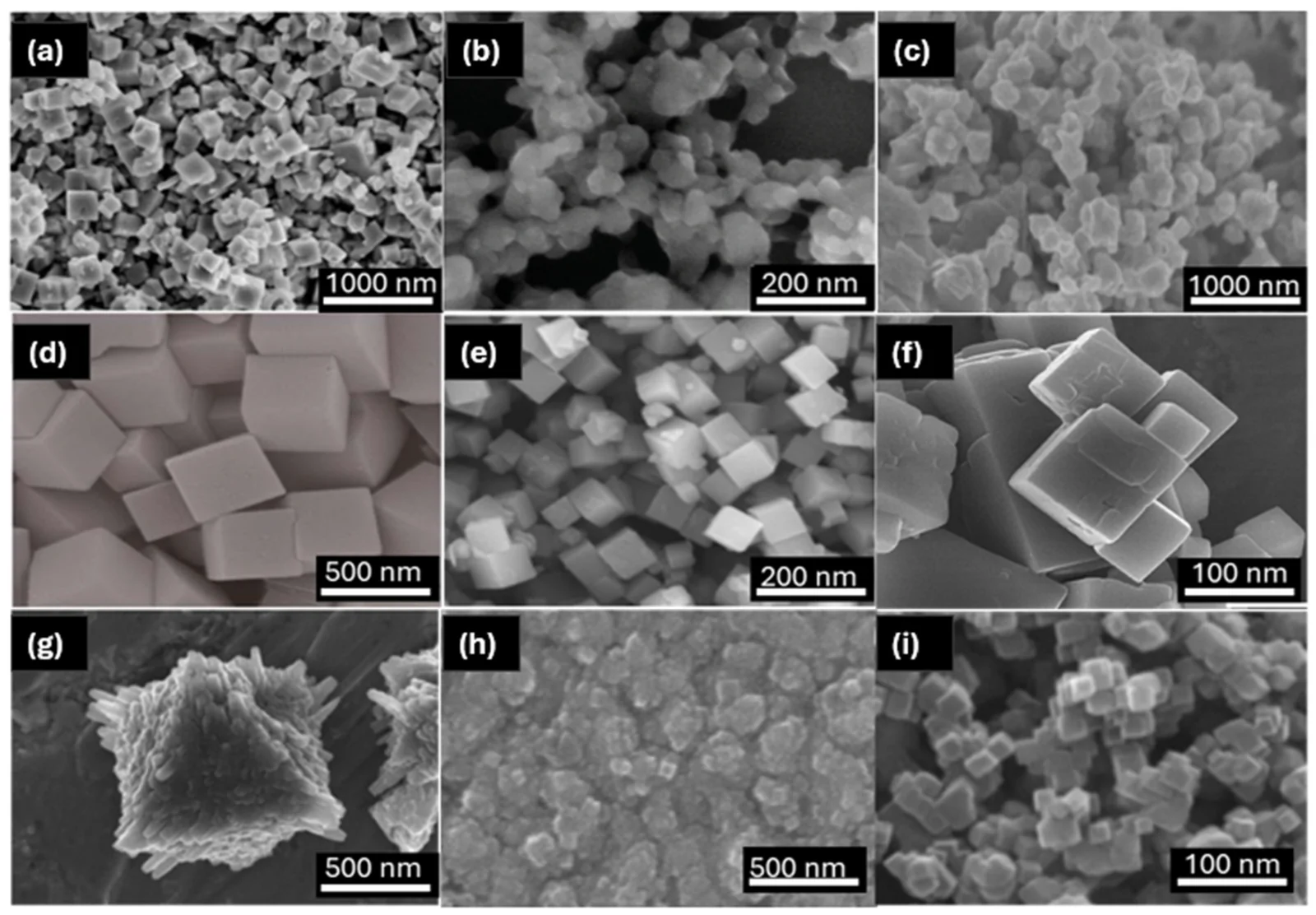
Representative SEM images of PBAs synthesized via different routes, (**a**–**c**) PBAs obtained by coprecipitation, showing irregular and aggregated particles with a wide size distribution. Based on [70,73,93]. (**d**–**f**) PBAs with a well-defined cubic morphology, associated with controlled synthesis conditions (optimized or hydrothermal coprecipitation). Based on [78,94]. (**g**) Hierarchical “flower-like” structures, indicative of self-assembly processes [76]. (**h**) Thin film obtained by electrodeposition, with uniform coverage on the substrate. Based on [95]. (**i**) PBA synthesized by ball milling, characterized by agglomerated nanoparticles and an absence of defined facets. Based on [88].

The work of Lan Li et al. [73] provides clear evidence of the impact of the coprecipitation method through SEM analysis, in which cubic particles with a wide size distribution are observed, coexisting with irregular aggregates and poorly defined edges (Figure 4a). This morphology is consistent with a rapid nucleation and uncontrolled growth regime, where the simultaneous formation of multiple nuclei limits the evolution toward well-faceted structures. Similarly, the study by Wessells et al. [70] reports highly aggregated NiHCF nanoparticles (Figure 4b), demonstrating that conventional coprecipitation favours coalescence and the formation of disordered structures. Likewise, Yanhong Ding et al. [93] the SEM analysis of the samples [93] reveals structures with heterogeneous size distribution and limited geometric definition (Figure 4c), reinforcing the idea that synthesis under suboptimal conditions leads to materials with low morphological uniformity.

Both Lan Li and Wessells et al. [70,73] agree that structural disorder due to rapid nucleation is the dominant factor in the process, also highlighting that subsequent aggregation plays a critical role in the loss of structural definition and the evolution toward less controlled morphologies. Yanhong Ding et al. [93] further support this view by showing that small variations in synthesis conditions can amplify morphological heterogeneity. Based on the findings of Lan Li, Wessells, and Ding et al. [70,73,93] a clear convergence is observed regarding a relevant structural limitation that is often underestimated in the discussion. In particular, the lack of control over nucleation kinetics not only affects particle size uniformity but also directly impacts key parameters, such as the accessibility of active sites and ion transport in the crystal lattice.

However, the literature also reports coprecipitation synthesis methods with greater parameter control, allowing for the development of more defined morphological structures. In this regard, You et al. [78] report obtaining highly defined nanocubes (Figure 4d) through precise control of pH and addition rate, while Sterzinger et al. [94] demonstrate that the incorporation of modulating agents allows for a reduction in defect density and the production of particles with greater morphological uniformity (Figure 4e).

Following this synthesis approach, You et al. [78] reports the formation of highly uniform nanocubes (Figure 4f), with well-defined faces and narrow size distributions, demonstrating a much more ordered morphology compared to materials obtained by conventional coprecipitation. Similarly, Hu et al. [76] show, using SEM, the formation of mesocrystals with a hierarchical organization (Figure 4g), suggesting not only optimization and better control of growth, but also structural self-assembly processes. Obtaining this structure is advantageous for combining high surface exposure with an open diffusion network.

On the other hand, Hedley et al. [95], which used the sequential electrodeposition method for the formation of PBA thin films on FTO conductive substrates (Figure 4h), achieved well-defined structures composed of FeCr-PBA cubic crystals in a first layer followed by the growth of a second PBA phase with nanometric domains. SEM provides evidence of coating continuity, spatial homogeneity, and relative thickness.

Among other studies, the mechanochemical pathway reported by Cheng et al. [88] stands out, using the ball milling method to reduce interstitial water content. SEM images (Figure 4i) reveal irregular particles, but with a tendency towards denser, packed structures. Unlike coprecipitation or hydrothermal processes, where nucleation and crystal growth are controlled, ball milling does not produce directional crystal growth; instead, it generates nanostructured domains that tend to aggregate spontaneously, forming compact secondary structures.

The various synthetic approaches mentioned regarding the morphology of PBAs demonstrate that their structure is a functional parameter of the material’s electrochemical performance. Disordered structures with high size dispersion and aggregation, a larger specific surface area, and a higher defect density are advantageous for applications such as electrochemical sensors, electrocatalytic materials, supercapacitors or pseudocapacitive storage, batteries, and adsorption and contaminant removal systems. However, it should be considered that a significant fraction of the material may not be truly available to the electrolyte due to stacking or aggregation, local compaction, or partial obstruction of surface channels.

Conversely, PBAs with highly defined morphologies and low defect density favour applications requiring high, long-term structural stability and controlled ion transport, such as batteries and ion intercalation, stable electrocatalysis under extended operation, electrochromic devices, or systems where selectivity is important. In addition to morphological analysis, it is essential to address the micro- and nanostructural differences in crystalline organization, where X-ray diffraction (XRD) is a key tool for delving into intrinsic structural aspects that are not accessible through morphological analysis or imaging techniques.

Through XRD analysis, it is possible to corroborate the characteristic patterns of PBAs with well-defined reflections associated with crystallographic planes (200), (220), (400), and (420). This technique is useful for evaluating the incorporation of cations with different ionic radii, since these changes cause a shift in the diffraction peaks, revealing modifications in the lattice parameters. PBAs are characterized by having high structural tolerance, which allows them to maintain their overall crystalline phase even at high defect densities.

Through XRD analysis, subtle deviations from the ideal structure can be detected by observing variations in the position, intensity, and width of the peaks. These variations can be associated with expansion or contraction phenomena in the unit cell, the appearance of secondary phases, or symmetry changes due to hydration. These structural variations are critical in PBAs, as they directly influence ionic mobility, structural stability, and, consequently, the electrochemical performance of the material.

In this context, studies reported in the literature establish clear relationships between the synthesis process and the evolution of the crystalline structure, demonstrating how small variations in composition or processing can translate into significant changes in lattice parameters and material behaviour.

**Figure 5:**
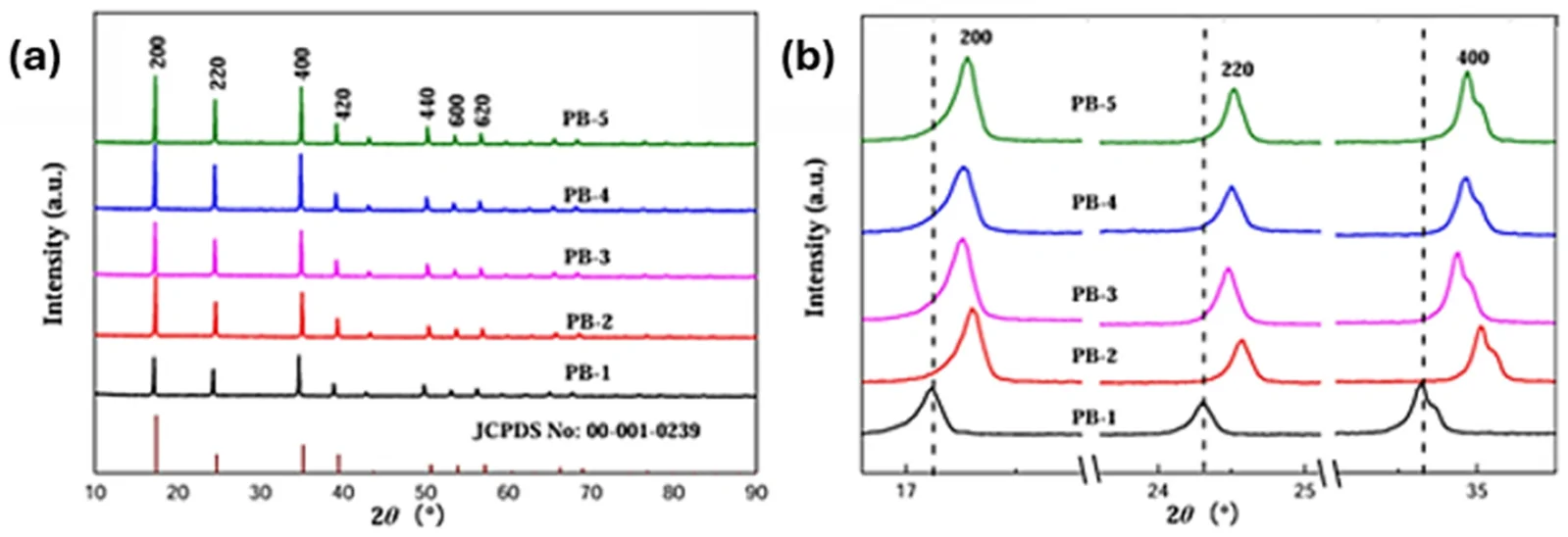
(**a**) X-ray diffraction patterns, (**b**) XRD patterns magnified between 16° and 36° of all samples. Based on Lan Li et al. (2019) [73].

Among the reported works, the research by Lan Li et al. [73] confirms the formation of PBA with high sodium content (up to Na_3.92_ per formula unit) along with a marked iron deficiency, demonstrating the presence of cationic defects in the lattice. The diffraction patterns (Figure 5) confirm that the synthesized material retains the Fm-3m cubic structure, despite the high concentration of defects and the presence of sodium, thus demonstrating the remarkable structural tolerance of the lattice to compositional perturbations. The same analysis shows a shift in the peaks towards larger diffraction angles, indicating a contraction of the unit cell associated with the decrease in Fe content.

**Figure 6:**
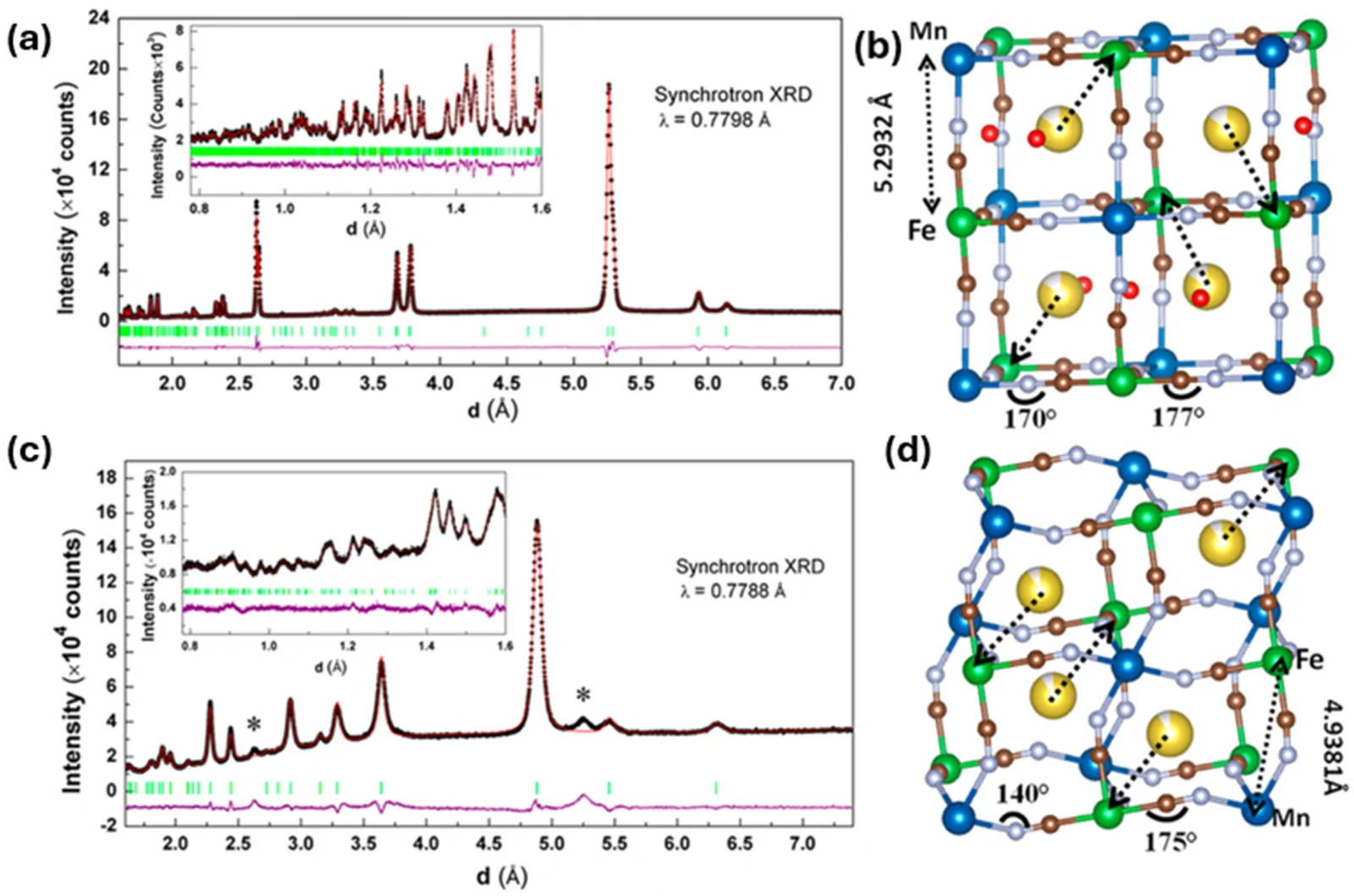
Synchrotron X-ray diffraction (SXRD) patterns of: (**a**) M-Na_2_−δMnHCF and (**c**) R-Na_2_−δMnHCF. Local structures: (**b**) M-Na_2_−δMnHCF and (**d**) R-Na_2_−δMnHCF, showing Na^+^ displacements and structure distortion. Asterisks (*) in panel (**c**) indicate peaks arising from absorbed water during sample preparation for SXRD. Based on Song et al. (2015) [96].

Similarly, the study by Song et al. [96] on the characterization of synthesized PBA highlights the close relationship between composition, structural stability, and interstitial water content. Elemental analysis (ICP) confirms a stoichiometry close to Na_1.89_Fe_0.97_Mn_1.00_(CN)_6_·zH_2_O, indicating a slight iron deficiency in the lattice, consistent with the presence of vacancies. SXRD analysis (Figure 6) reveals a structural transition induced by the removal of interstitial water. The hydrated material exhibits a monoclinic structure (P2_1_/n), while the dehydrated phase adopts a rhombohedral structure (R$\bar{3}$), demonstrating the direct influence of water on crystal symmetry. Furthermore, the diffraction pattern shows a peak marked with an asterisk, attributable to secondary phases, possibly due to the presence of impurities or partial transformations of the material, either through decomposition or oxide formation during synthesis. These crystal lattice transformations, associated with cooperative displacement between cations and rotation of cyanide bonds, modify the distances between the transition metal and iron, thus altering the unit cell volume, making it more compact and distorted. This behaviour confirms that PBAs have a structure that is not rigid but highly adaptable to chemical perturbations.

**Figure 7:**
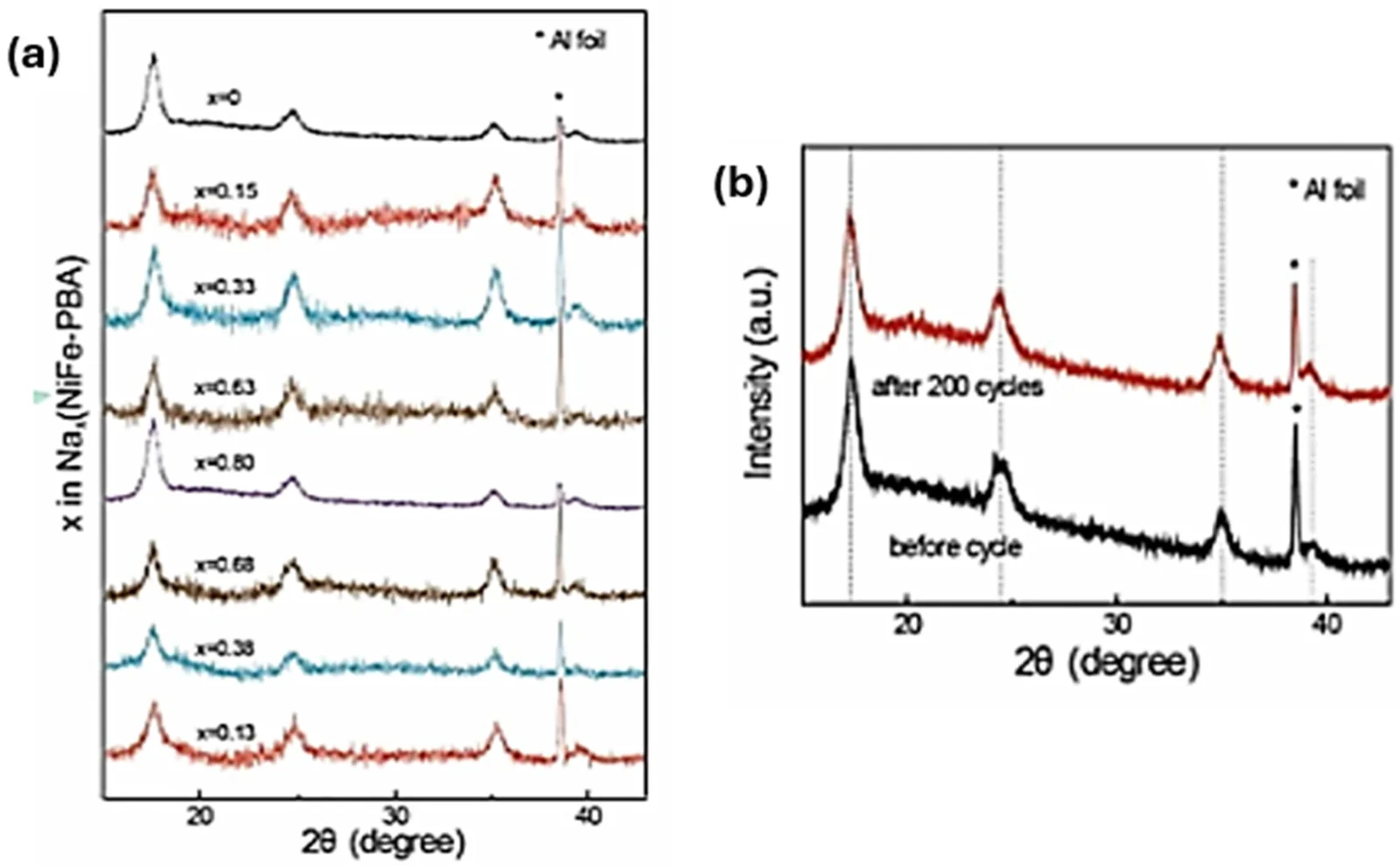
Characterization of ex situ X-ray diffraction patterns (**a**) of NiFe-PBA cathodes, (**b**) before and after a 200-cycle charge–discharge process. Based on Yang et al. (2022) [97].

Furthermore, Yang et al. [97] reported and discussed previous studies on monovalent (Na and K) and multivalent metal (Mg, Ca, Zn, and Al) PBAs, in which XRD analysis was used to validate the formation of PBA structures and demonstrate the effect of incorporating cations with different ionic radii. The diffraction patterns (Figure 7a) show the formation of the typical PBA phase, where a shift in the (20°) peak towards larger angles can be observed, indicating a contraction of the lattice parameter attributed to the incorporation of K^+^ and, consequently, the volumetric reduction in the unit cell. Meanwhile, Figure 7b shows a comparison of the diffraction patterns before and after charging and discharging tests. After 200 electrochemical cycles, the main reflections of the PBA remain in similar positions, confirming structural stability during the process. However, there are changes in intensity and a slight broadening of the peaks, suggesting a progressive generation of structural disorder, attributable to defect formation, cationic redistribution, or hydration. These results indicate that PBAs exhibit high structural stability; however, they can undergo local modifications in the crystal lattice due to prolonged cycling processes, which could gradually impact electrochemical performance.

Furthermore, the work of Wessells, You, and Pasta et al. [70,78,82] demonstrates that the XRD analysis performed by each of them shows a similarity in confirming the face-centred cubic (FCC) structure, with reflections of (200), (220), (400), and (420), but with critical structural differences associated with the synthesis method and crystal quality.

**Figure 8:**
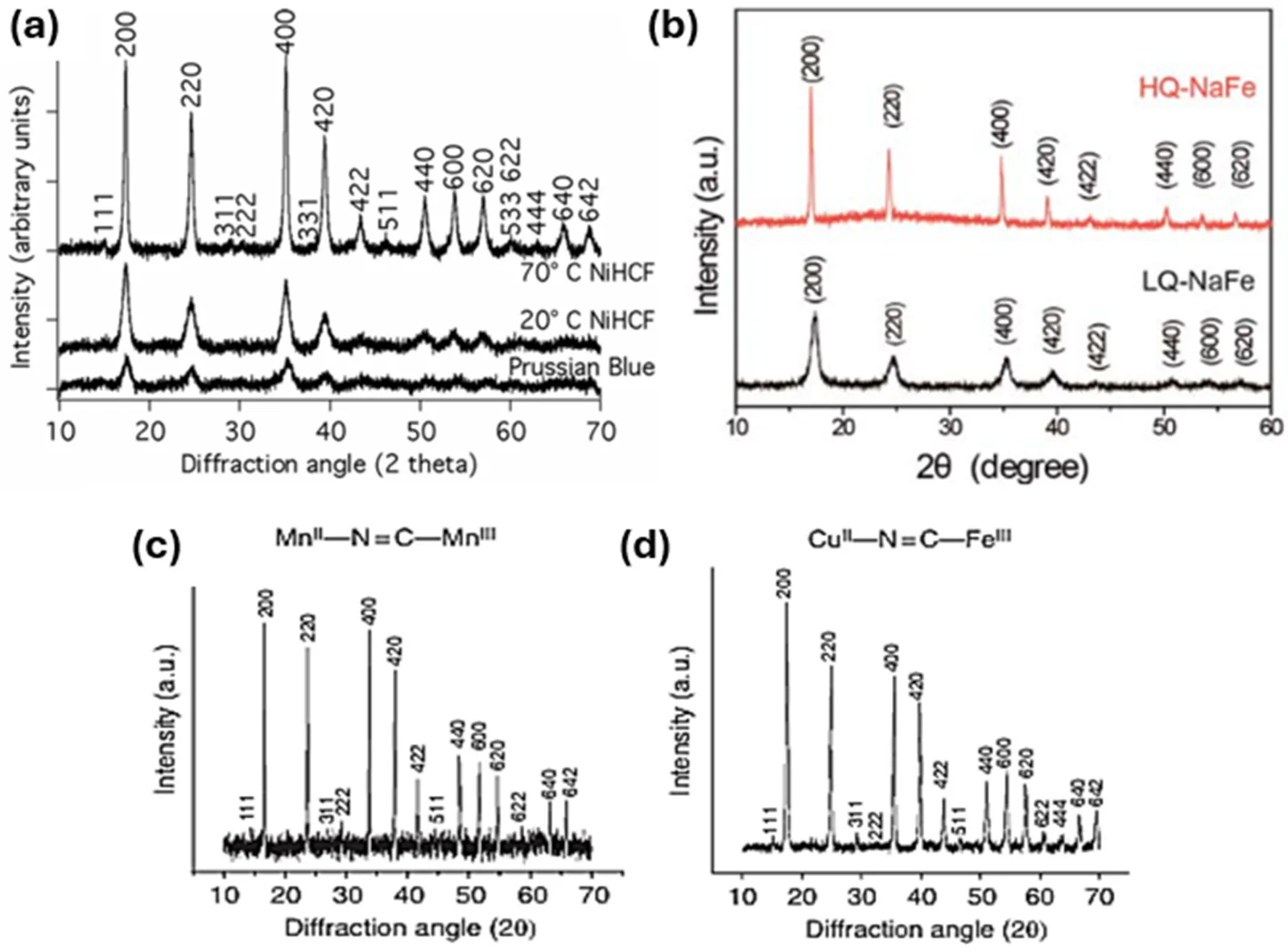
X-ray diffraction patterns of different PBAs and their structural variations. (**a**) Comparison between NiHCF synthesized at 20 °C and 70 °C, along with the Prussian blue reference. Based on Wessells et al. (2011) [70]. (**b**) Diffractions of NaFe with high (HQ) and low (LQ) quality. Based on You et al. (2014) [78]. (**c**) XRD pattern of Mn–N≡C–Mn and (**d**) XRD pattern of Cu–N≡C–Fe. Based on Pasta et al. (2014) [82].

Wessells et al. [70] show the difference that exists in the diffraction patterns (Figure 8a) when the material is synthesized at different temperatures, favouring crystal growth. A material obtained by hydrothermal methods will have more defined crystals, increasing the degree of crystallinity, which is reflected in more intense and narrower peaks. In contrast, the lower-temperature synthesis exhibits broadened peaks of lower intensity, suggesting the presence of smaller crystalline domains and a greater degree of structural disorder.

You et al. [78] further explored the structure-synthesis relationship by directly comparing two synthesized materials of high quality (HQ-NaFe) and low quality (LQ-NaFe). XRD (Figure 8b) shows the peaks for both materials at the same angle, confirming the cubic phase, but with a clear difference between the HQ material, which has more intense and narrower peaks, indicating a higher degree of crystallinity and less structural disorder, and the LQ material, which exhibits broadened peaks of lower intensity, demonstrating a lower degree of crystallinity. Another important point is the slight variation in the lattice parameter (10.21 Å vs. 10.27 Å), which translates to an expansion of the unit cell due to these structural defects.

In the case of Pasta et al. [82], their approach is directed toward a comparative analysis between different PBAs (CuHCF and MnHCMn). The XRD (Figure 8c,d) demonstrates that both materials possess a cubic structure, with well-indexed reflections, confirming the robustness of the structure for different PBA compositions. The highlight of this research is the use of in situ XRD during redox processes, demonstrating that there are no significant phase changes, but there are slight peak shifts to larger angles associated with variations in the lattice parameters, mainly attributable to the decrease in the ionic radius of the metal centre due to the change in oxidation state. Thus, the direction of peak shift in XRD provides valuable information for distinguishing between structural defects and changes in the electronic state of the metals.

Taken together, these three studies demonstrate that XRD in PBAs fulfils multiple critical functions, including confirming the crystalline phase, evaluating crystallinity, and identifying defects based on peak width and intensity. However, it must be used with caution and complemented with other techniques to avoid misinterpreting the results. The direct attribution of changes in peak width and intensity to crystal size, deformation, and the presence of vacancies must be disaggregated or each contribution decoupled. In this sense, XRD alone is limited because it captures a structural average of the heterogeneities inherent in PBAs.

For a more robust interpretation, it is necessary to delve deeper into the surface chemistry and the electronic environment of the metal centres present in PBAs. In this regard, X-ray photoelectron spectroscopy (XPS) is relevant due to the coexistence of different oxidation states at the metal centres and the surface’s sensitivity to defects, vacancies, hydration, or chemical reconstruction processes. Through high-resolution spectra, specifically in regions such as Fe 2p, M 2p, C 1s, and N 1s, it is possible to identify metal cyanide coordination, distinguish oxidized or reduced species, and evaluate how the surface composition is modified by synthesis or electrochemical processing.

**Figure 9:**
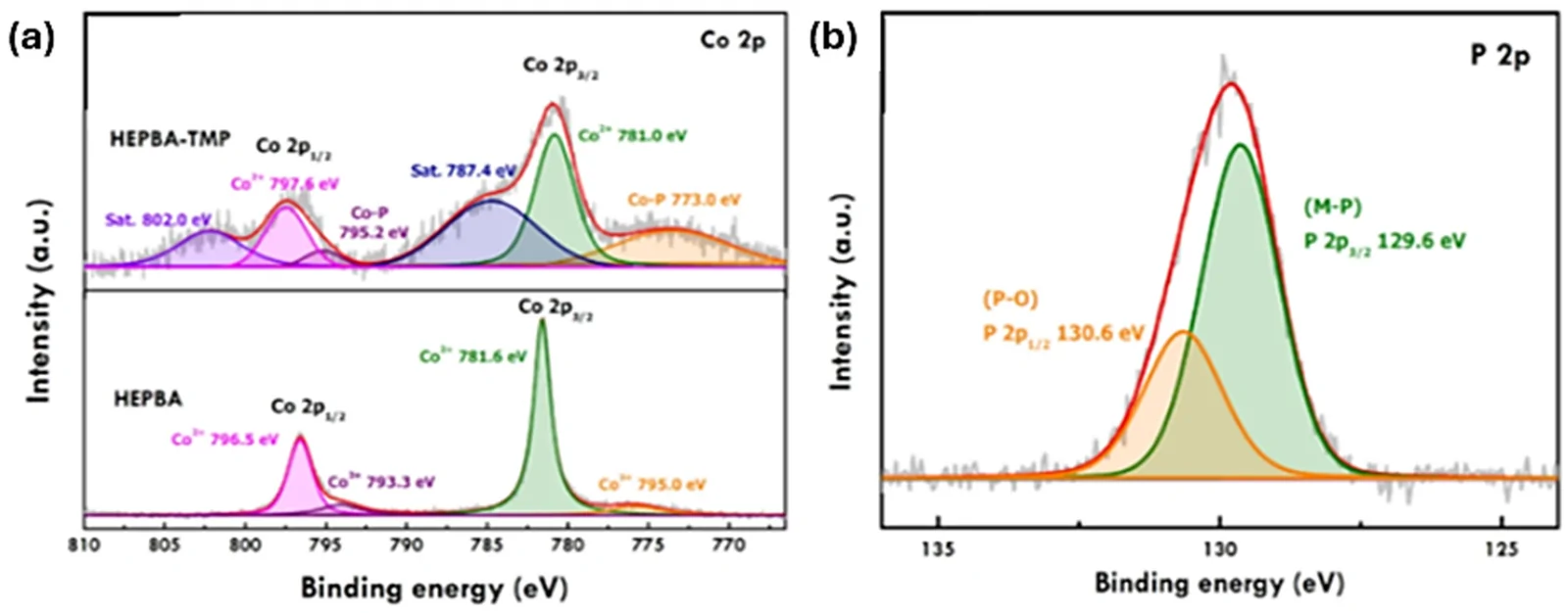
High-resolution XPS spectra of (**a**) Co 2p and (**b**) P 2p for HEPBA and its phosphorylated derivative HEPBA-TMP. Based on Liu et al. (2026) [91].

In this way, Liu et al. [91] used XPS analysis to evaluate the oxidation states between the precursor PBA (HEPBA) and its phosphorylated derivative (HEPBA-TMP). The results show the effective incorporation of phosphorus into the structure (Figure 9a), evidenced by the appearance of a signal in the P 2p region centred at 129.6 eV, attributed to the metal-phosphorus (M-P) bond. In the Co 2p region, the precursor material is predominantly Co^2+^, while after phosphorization, an electronic redistribution occurs, leading to the formation of mixed Co^1+^/Co^2+^ states, along with the appearance of characteristic Co-P bond signals. Similarly, the spectra of Ni 2p, Cu 2p, and Mn 2p show the coexistence of phosphorus-bonded metal species (Ni-P, Cu-P, Mn-P) with surface oxidized species (M-O), suggesting a chemically heterogeneous surface. Additionally, Figure 9b shows a peak centred at 129.6 eV, characteristic of metal-phosphorus bonds (M-P), along with the higher-energy contribution (130.6 eV) of P-O oxidized species. These results provide evidence that phosphorylation of PBA induces a structural transformation and electronic redistribution through the generation of active M-P bonds.

**Figure 10:**
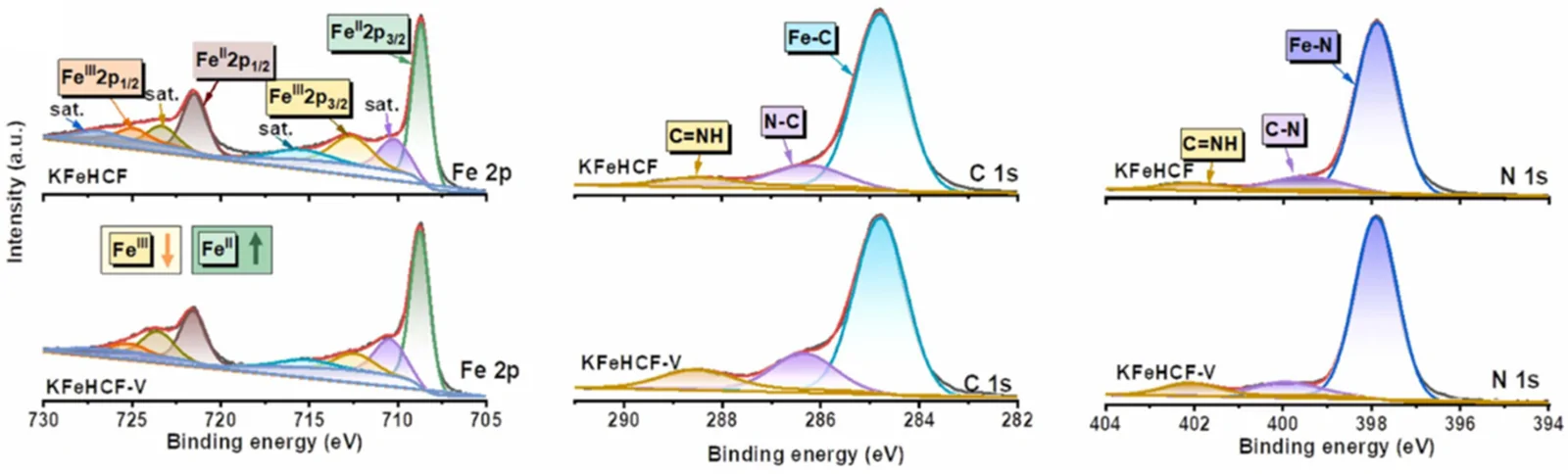
XPS spectra of Fe 2p, C 1s, and N 1s of KFeHCF and KFeHCF-V. Based on Wang et al. (2022) [98].

The study by Wang et al. [98] demonstrates how the introduction of Fe^3+^ vacancies in PBAs impacts the stoichiometry, crystal structure, and electronic state of the material. The EDS + ICP-MS analysis shows an increase in the K/Fe ratio for the KFeHCF-V material, reaching values close to K_1.74_FeFe(CN)_6_ compared to K_0.98_FeFe(CN)_6_ in the unmodified material. This increase in potassium is interpreted as a charge compensation mechanism due to the Fe^3+^ vacancies, confirming the formation of cationic defects within the lattice. XPS analysis (Figure 10) demonstrates the coexistence of Fe^2+^ and Fe^3+^, but with a decrease in the intensity of the signals associated with Fe^3+^ in the KFeHCF-V material, indicating an effective reduction in the oxidation state. Furthermore, the analysis confirms the spectra of C≡N≡C and N≡C in coordination, thus confirming the chemical stability of the bond network despite the incorporation of vacancies.

**Figure 11:**
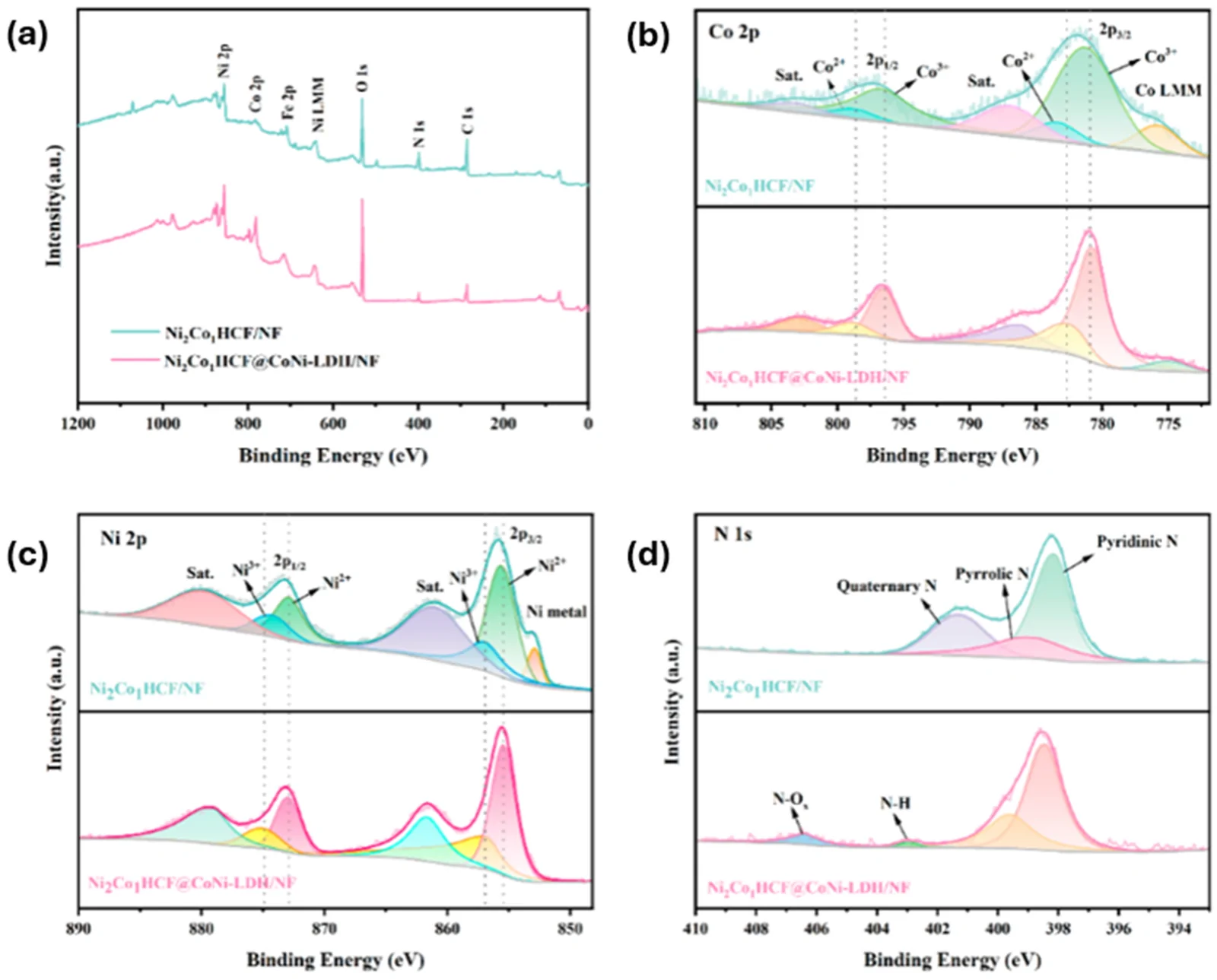
XPS spectra of (**a**) overall spectrum; (**b**) Co 2p peak; (**c**) Ni 2p peak; (**d**) N 1s peak of the compounds Ni_2_CoHCF/NF and Ni_2_CoHCF@CoNi-LDH/NF. Based on Yin et al. (2023) [99].

The work of Yin et al. [99], which uses electrodeposition for the synthesis of PBA, was complemented with XPS analysis data to confirm the surface composition, oxidation states, and electronic interactions in the Ni_2_CoHCF/NF system and its composite derivative Ni_2_CoHCF@CoNi-LDH/NF. Figure 11a confirms the presence of all constituent elements (Ni, Co, Fe, C, N, and O), supporting the correct formation of both the PBA and the composite material. For synthetic engineering, this result is important because it validates the electrodeposition process by not altering the chemical integrity of the PBA structure, even when an additional phase is introduced onto its surface. Figure 11b,c show the high-resolution analysis, where Co2p is identified with simultaneous contributions from Co^2+^ and Co^3+^, while in Ni2p mixed Ni^2+^/Ni^3+^ states are observed, confirming the redox-active nature of the material. Another important result is the shift in bond energies, with a decrease in values (0.3–0.6 eV) in the composite material compared to the original PBA, suggesting electron transfer between the Fe–Co–Ni centres. This effect is key, as it indicates a strong interfacial interaction between the PBA and the CoNi-LDH layer, through a modification in electron density that may favour electrochemical kinetics. Furthermore, Figure 11d shows the presence of species (pyridine, pyrrole, and quaternary nitrogen) in the PBA, while in the composite material, new contributions associated with N-H bonds and oxidized species (NOx) emerge, suggesting a chemical modification of the surface environment after electrodeposition. Therefore, the XPS analysis not only confirms the composition but also reveals a key electronic and interfacial reconfiguration of the PBAs.

**Figure 12:**
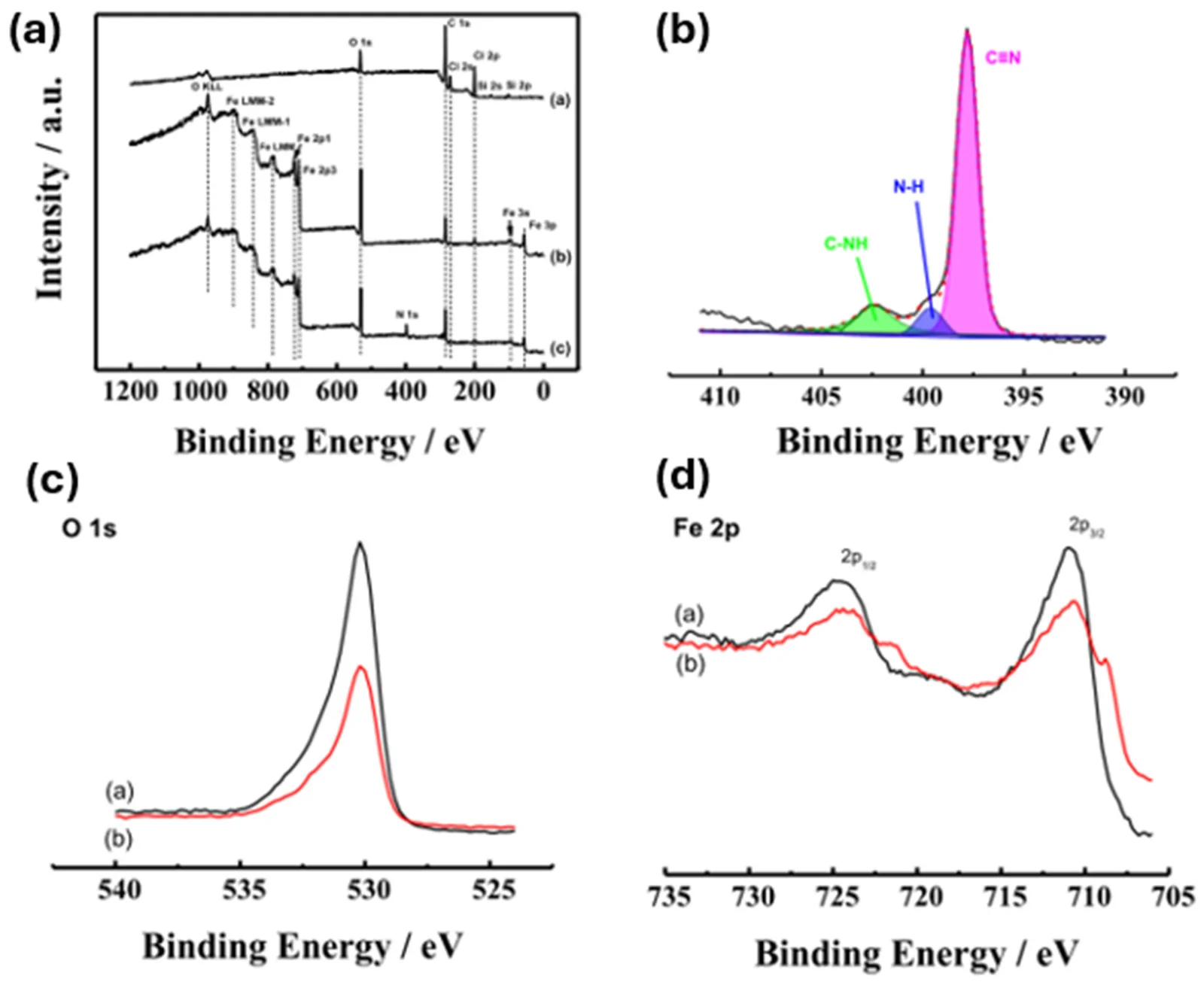
X-ray photoelectron spectrum: (**a**) Broad XPS spectrum of bare SPCE, SPCE/nano-Fe_3_O_4_ and SPCE/nano-Fe_3_O_4_/PBNC. (**b**) Narrow XPS spectrum of N1s from SPCE/nano-Fe_3_O_4_/PBNC. (**c**) Narrow XPS spectrum of O1s and (**d**) Fe2p from SPCE/nano-Fe_3_O_4_ and SPCE/nano-Fe_3_O_4_/PBNC. Based on Tse et al. (2022) [100].

In this same scenario, Tse et al. [100] performed electrodeposition of PBA on a modified substrate (Fe_3_O_4_/C). XPS analysis confirms the presence of characteristic elements along with substrate-related signals (Figure 12a) and the characteristic Fe–C≡N–Fe framework formation, through N 1s signals associated with the cyanide bond and showing additional contributions linked to species in the medium (glyphosate), a key point as it suggests chemically assisted growth (Figure 12b). Furthermore, Figure 12c,d shows the differences in intensities associated with the O 1s and Fe 2p spectra, highlighting the changes in the chemical environment of oxygen, due to the coating itself or to assumed contributions (adsorbed water, hydroxyl groups, oxides), and the different oxidation states of iron (Fe^2+^/Fe^3+^), consistent with the mixed nature of PBAs. However, the lack of detailed signal reconstruction hinders a precise identification of the electronic state distribution.

The work of Yin and Tse et al. [99,100] complements each other and demonstrates different approaches to the synthesis method, which are directly reflected in the scope of XPS analysis. For Yin et al. [99], electrodeposition is a tool in interfacial engineering for constructing PBA@LDH heterostructures, where XPS allows the identification of mixed oxidation states and shifts in bond energy that demonstrate charge transfer between phases. In contrast, Tse et al. [100] employ a coating method where XPS plays a verifying role regarding the presence of the C≡N bond and the incorporation of electrolyte species, without delving into the nature of the interface or electronic effects. XPS can be used as a tool for compositional validation and mechanistic interpretation, demonstrating the true potential of the PBA synthesis method, as it lies not only in the formation of the material but also in the modulation of its electronic structure and functionality.

In addition to XPS analysis, vibrational techniques such as Raman and FTIR are necessary to further investigate the nature of the bonds and the local structure of PBAs. In this context, these techniques allow for the direct resolution of the characteristic vibrations of the C≡N group, as well as the detection of changes in metal-ligand coordination (M–C≡N–M’), the presence of interstitial water, and possible structural modifications induced by synthesis or electrochemical conditions. Through a complementary approach, the electronic structure can be correlated with bond chemistry, leading to a more comprehensive understanding of PBA behaviour.

**Figure 13:**
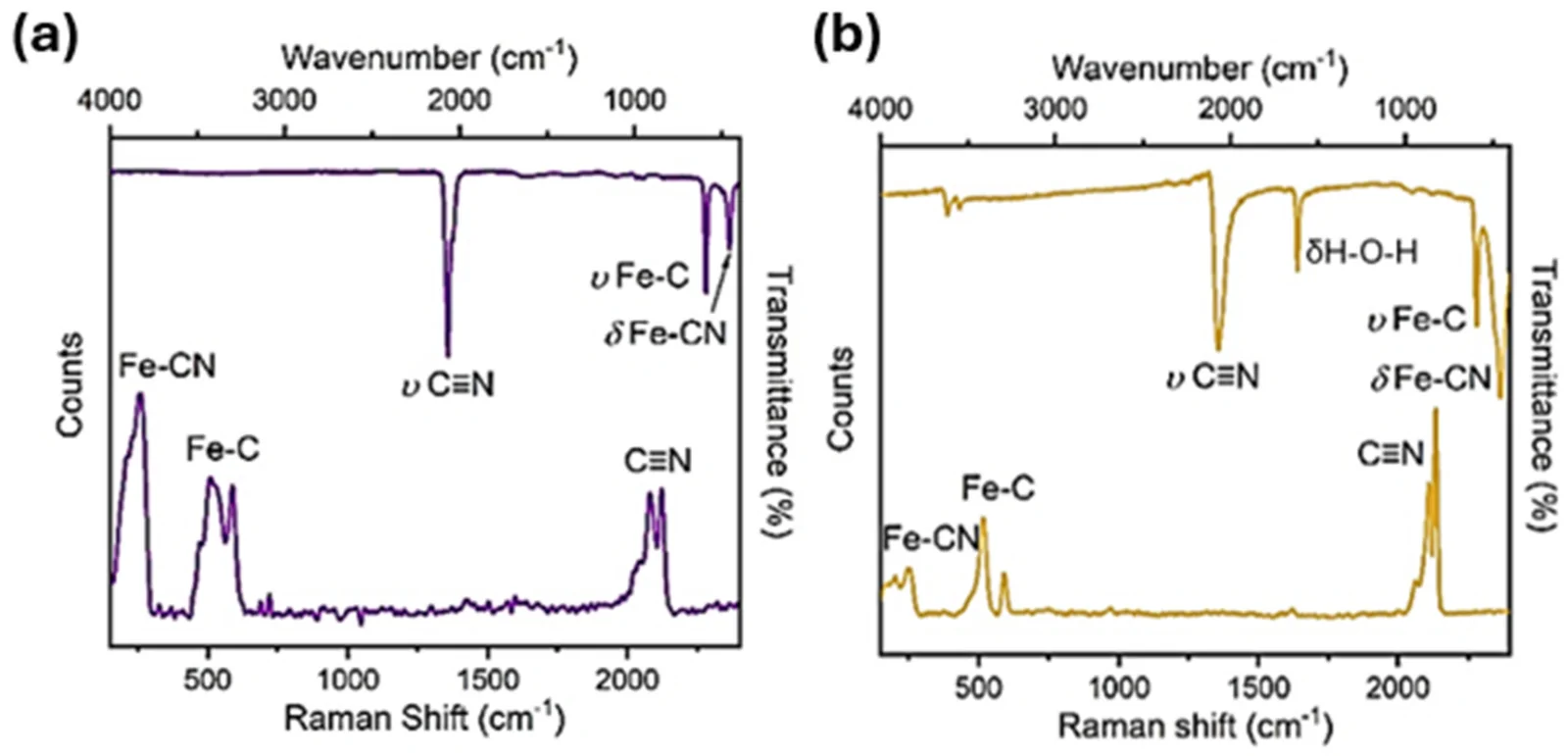
FT-IR and Raman spectra of (**a**) KPB and (**b**) NaPB. Based on Tinker et al. (2024) [101].

In this way, the work of Tinker et al. [101] on obtaining PBA by coprecipitation for sodium ion storage applications was supported by FTIR and Raman vibrational techniques to characterize the local structure and coordination environment with different alkali cations (K^+^ and Na^+^) (Figure 13). Based on FTIR analysis, both materials (KPB and NaPB) exhibit the characteristic bands of the HCF structure, the ν(C≡N) stretching vibration (2065 cm^−1^), as well as those associated with ν(Fe–C) and δ(Fe–C–N) strains. A key finding is the shift to lower wavenumber values of the C≡N bond corresponding to the material with the higher water content (NaPB), supported by the more intense presence of the δ(H-O-H) vibrational band (1615 cm^−1^). This result indicates that interstitial hydration modifies the electronic environment of the C≡N bond, partially weakening its vibrational rigidity. Raman analysis, for its part, allows for a more in-depth structural interpretation by clearly identifying the Eg and A1g modes of the C≡N group (between 2000 and 2200 cm^−1^), whose position depends on the oxidation state of iron (Fe^2+^/Fe^3+^). In this regard, a shift to higher wavenumbers is observed in NaPB compared to KPB, indicating a higher average oxidation state of Fe and a stronger Fe–CN interaction. However, interstitial hydration and structural vacancies can also influence the rigidity of the CN bond. In the low-frequency regions (450–620 cm^−1^ and 190–340 cm^−1^), Fe–C vibrations and Fe–CN–Fe deformations occur, confirming the structural integrity of the PBA.

**Figure 14:**
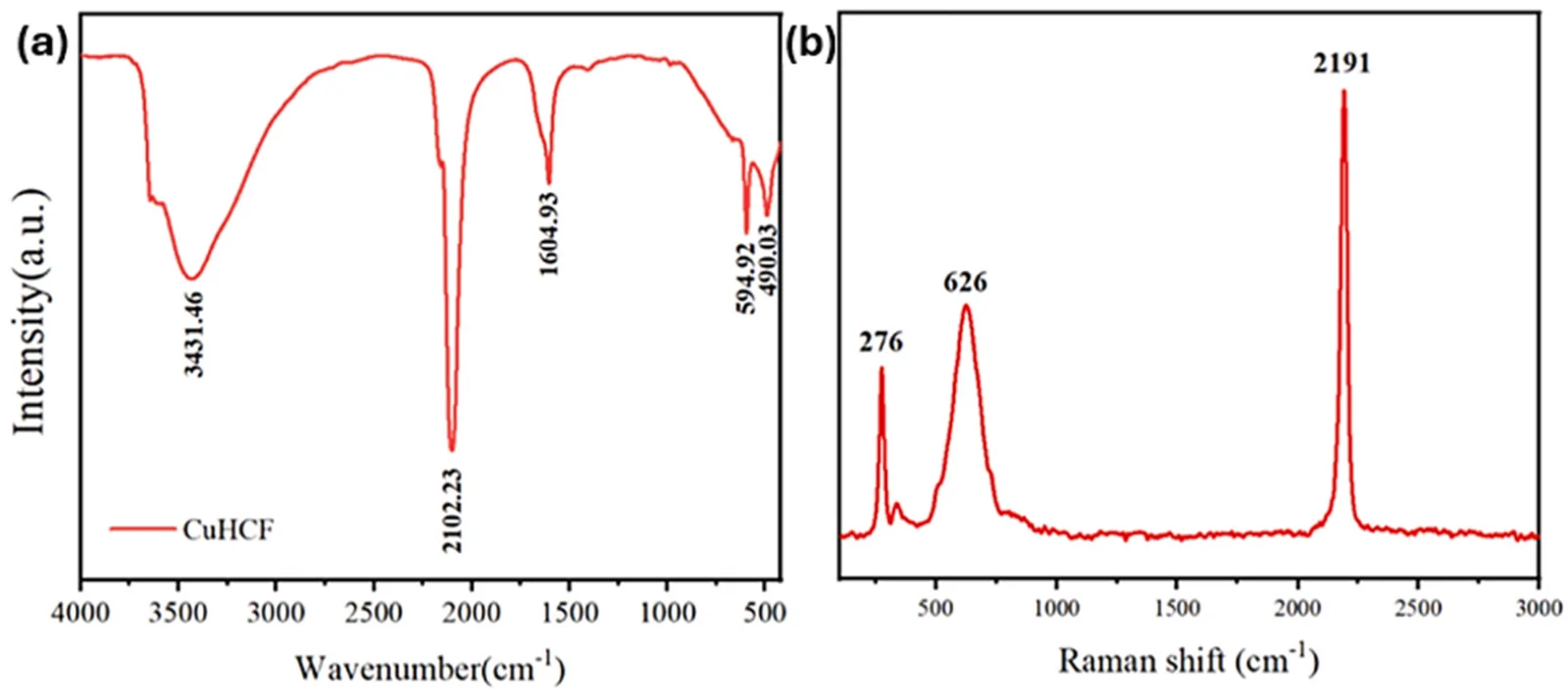
(**a**) FTIR spectra and (**b**) Raman spectra of CuHCF. Based on Zeng et al. (2024) [23].

In the work of Zeng et al. [23], FTIR and Raman techniques were used to confirm the formation of CuHCF and its local structure. The FTIR analysis (Figure 14a) identifies the characteristic vibrations of the PBA structure, with the 2102 cm^−1^ band attributed to the Fe^3+^–C≡N–Cu^2+^ bond, confirming the correct formation of the material. It also shows bands at 1605 cm^−1^ and 3431 cm^−1^ corresponding to δ(H–O–H) and ν(O–H), respectively, which demonstrate the presence of interstitial water. Thus, the analysis confirms the presence of the cyanide group and the interstitial water content, key aspects in the impact of ion transport and structural stability. Furthermore, Raman analysis (Figure 14b) complements the structural information by resolving the vibrational modes of the material’s structure with greater precision. The main band at 2191 cm^−1^, associated with the stretching of the C≡N triple bond, confirms the presence of the cyanometallic framework. Additionally, the signals at 626 cm^−1^ and 276 cm^−1^, assigned to Fe–C vibrations and Fe–CN–Fe bridge deformations, respectively, demonstrate the integrity of the crystal lattice.

The work of Tinker and Zeng et al. [23,101] using FTIR and Raman spectroscopy, provides information for characterizing PBAs by probing the environment of the C≡N bond, which constitutes the structural core of these materials. The sensitivity of the FTIR signals confirms the presence of interstitial water and functional groups, allowing for the evaluation of the degree of hydration and its potential impact on stability and ion transport. Raman analysis, on the other hand, provides higher resolution of the vibrational modes of the crystal structure and can detect variations in metal-ligand coordination and the electronic state of the metal centres. The authors used these analyses to confirm and correlate the local structure, hydration, and electrochemical properties of PBAs.

The characterization of PBAs necessarily requires a comprehensive approach, as no single technique can capture the structural and chemical complexity of these materials. This is evidenced by the results reported in the investigations during this chapter, with a strong emphasis on SEM analysis, which provides information on particle organization and the degree of aggregation, thus regulating the accessibility of the ion channels characteristic of these materials. Meanwhile, XRD is a key analysis for confirming the typical crystal structure of PBAs and evaluating how the lattice parameters are affected by the inclusion of defects, vacancies, or changes in the material’s stoichiometry. For its part, XPS allows for a deeper understanding of the oxidation states of the metal centres and the bond chemistry, providing information on the electronic environment that governs the material’s redox activity. In this context, vibrational techniques such as FTIR, designed to identify the presence of interstitial water and functional groups, and Raman, focused on detecting variations in C≡N bond stiffness associated with changes in electronic state, hydration, or the presence of defects, are integrated in a complementary way into the material analysis. This allows for the validation of the local C≡N bond structure and the characterization of the metal-ligand coordination environment.

The integration of all these techniques provides a solid foundation for understanding how different variables (defects, interstitial water, composition, and electronic environment) directly influence the material’s performance. This, in turn, advances the development of more rational design strategies aimed at adjusting the PBA structure according to its application, especially in electrochemical systems that require high stability and selectivity under demanding conditions.

## 6. Use of PBA in Seawater

The high concentrations of Cl^−^, as well as multivalent cations such as Mg^2+^ and Ca^2+^, present in seawater, represent a critical challenge for electrocatalysts used in seawater electrolysis [19,102]. These ions can induce multiple undesirable effects, such as localized corrosion, precipitation of poorly soluble salts, and the formation of byproducts due to competitive reactions, such as CER under anodic conditions. Furthermore, the adsorption of these species can block active sites on the catalyst, causing catalytic poisoning, which drastically reduces the efficiency of the target reaction [38,102,103,104]. In this context, PBAs have demonstrated a fundamental role due to their unique ability to act as redox active even under chemically aggressive conditions such as seawater, showing structural stability and resistance to corrosion induced by reactive species such as Cl_2_ or HOCl [105,106,107,108]. Several studies have demonstrated that these materials can maintain catalytic activity under non-ideal conditions, exhibiting relatively low overpotentials and good electrochemical stability in solutions with high chloride content. Their resistance to degradation and electrochemical performance position PBAs as attractive candidates for electrolysis applications with saline electrolytes, particularly where freshwater is scarce.

From a fundamental standpoint, the presence of high concentrations of chloride ions, along with multivalent cations such as Mg^2+^ and Ca^2+^, introduces several competing phenomena that directly impact catalyst performance. These include (i) competitive adsorption at active sites, (ii) precipitation of insulating hydroxides such as Mg(OH)_2_ and Ca(OH)_2_, and (iii) the promotion of parasitic anodic reactions such as the CER. As a result, the effective catalytic response in seawater cannot be evaluated solely based on intrinsic activity metrics but must instead be understood as a function of interfacial stability and reaction selectivity.

In this context, PBAs offer several potential advantages. Their open framework structure, combined with tunable metal centres and redox-active sites, provides a unique platform for modulating adsorption energetics and facilitating ion transport. In particular, the cyanide-bridged network can stabilize intermediate species while allowing dynamic structural adaptation under electrochemical polarization. This behaviour may contribute to mitigating surface poisoning and maintaining catalytic activity under high ionic strength conditions.

However, despite these advantages, current experimental evidence suggests that PBAs still lag behind state-of-the-art catalysts in terms of intrinsic activity. Reported overpotentials for HER in saline media, typically in the range of −0.75 to −0.85 V vs. SHE for systems such as NiHCF and CuHCF, remain significantly higher than those observed for benchmark catalysts such as NiMo or Pt-based materials. Similarly, for anodic processes, the competition between OER and CER is not fully suppressed, particularly under non-optimized conditions, raising concerns regarding selectivity and long-term stability.

A critical limitation in the current literature is the lack of standardized testing protocols. Many studies rely on simplified electrolyte compositions, often using artificial seawater or NaCl-based solutions, which fail to capture the full complexity of real seawater systems. Consequently, reported performance metrics are difficult to compare across studies, and may overestimate the practical viability of PBAs under industrially relevant conditions.

Furthermore, emerging evidence suggests that PBAs may not act as stable catalytic phases during operation, but rather as dynamic precursors that undergo surface reconstruction toward more active species, such as transition metal (oxy)hydroxides. In this scenario, the role of the PBA framework shifts from being the primary catalytic phase to acting as a structural and electronic scaffold that governs the formation and stabilization of the true active sites. This perspective aligns with recent trends observed in other electrocatalytic systems, where metastable or precursor phases play a critical role in determining catalytic performance.

Taken together, these findings indicate that while PBAs exhibit promising features for seawater electrolysis, their current performance remains limited by intrinsic activity, electronic conductivity, and insufficient control over interfacial processes. Future research should therefore focus on integrating PBAs within hybrid or heterostructured systems, enhancing electronic transport, and systematically evaluating performance under realistic seawater conditions [46,49,101,105,109,110,111].

### 6.1. NiHCF

NiHCF is among the most extensively studied PBAs in saline electrochemical systems, owing to its well-defined redox chemistry, structural stability, and versatility across applications ranging from ion storage to electrocatalysis. Its electrochemical behaviour is primarily governed by the reversible Fe^2+^/Fe^3+^ redox couple within the cyanide-bridged framework, which enables efficient charge transfer and ion intercalation. In seawater environments, these features translate into a material capable of maintaining electrochemical functionality despite the presence of competing ions and high chloride concentrations. However, its role as an electrocatalyst remains complex and cannot be fully understood without considering its dynamic structural evolution under operating conditions.

From a catalytic perspective, NiHCF has shown activity toward the OER in chloride-containing media, although its performance remains dependent on the electrolyte composition and the structural characteristics of the material. Beyond the overpotential, reaction selectivity becomes particularly relevant in saline environments, where OER directly competes with CER. In this regard, the hydration state of NiHCF has been identified as an important factor influencing the relative contribution of both reactions. Structural water, together with the local coordination environment of the Fe–CN–Ni framework, may modify the interaction of the surface with oxygenated and chloride species. Therefore, NiHCF is particularly interesting for studying how structural features of PBAs affect OER/CER selectivity under chloride-rich conditions [112].

**Figure 15:**
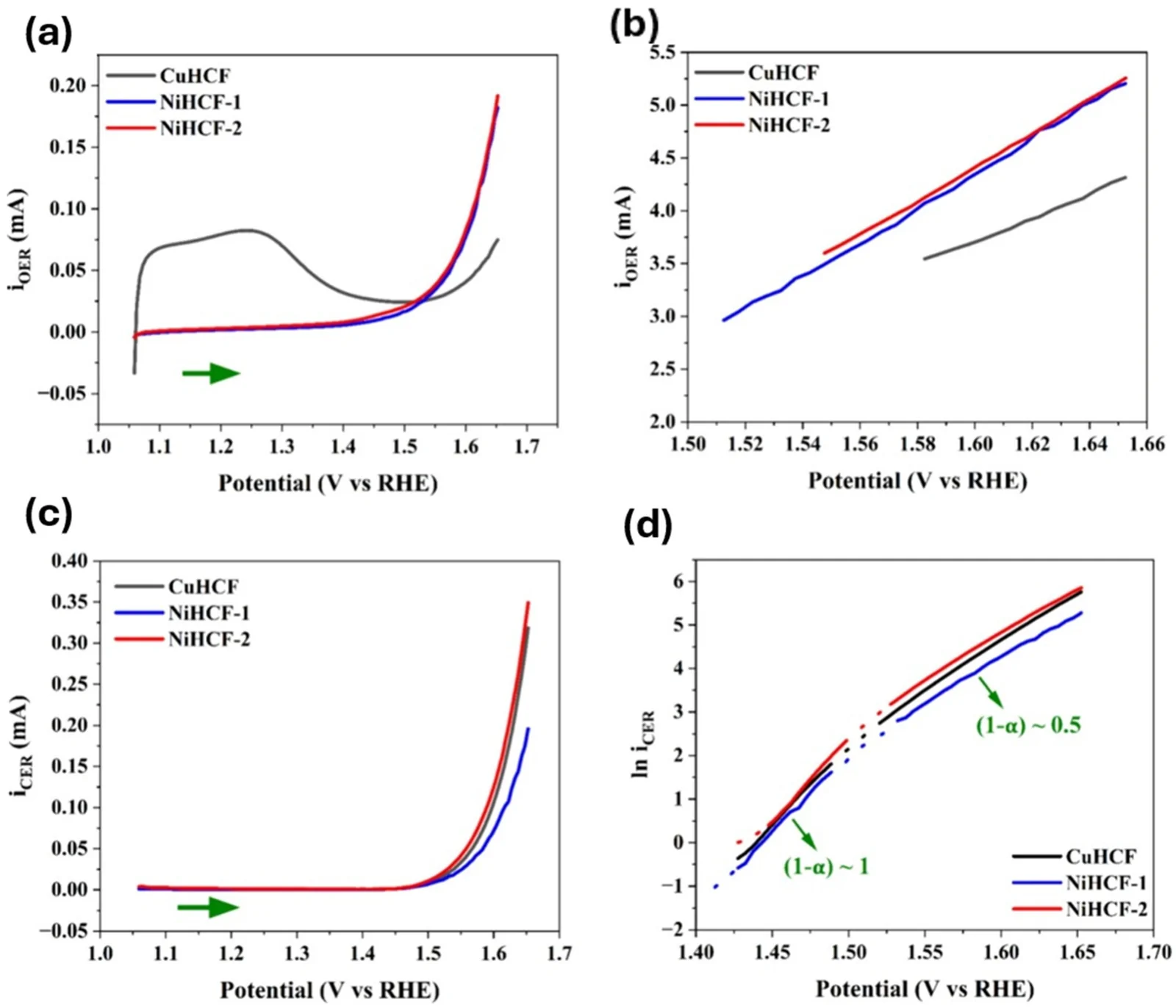
(**a**) Polarization curve (OER) of NiHCF by hydration method, (**b**) Corresponding Tafel curve plots, (**c**) Polarization curve (CER) of NiHCF by hydration method, (**d**) Corresponding Tafel curve plots. RRDE voltammetry was performed in an Ar-saturated electrolyte containing 0.5 M NaCl + 50 mM H_2_SO_4_ at a scan rate of 10 mV s^−1^ and an electrode rotation speed of 1000 rpm. Based on Raja et al. (2025) [112].

Raja et al. [112] evaluated the OER/CER selectivity of hydrated NiHCF and CuHCF electrocatalysts in an acidified chloride-containing medium (0.5 M NaCl + 50 mM H_2_SO_4_). Using rotating ring-disk electrode (RRDE) measurements, the authors were able to distinguish the contributions of oxygen and chlorine evolution to the overall oxidation current, as shown in Figure 15. NiHCF exhibited an OER overpotential of approximately 270 mV at 10 mA cm^−2^, together with a Tafel slope of 41.6 mV dec^−1^. More importantly, the OER selectivity depended strongly on the hydration state of the material, reaching 26.47% for NiHCF-1 and 14.56% for NiHCF-2, compared with 10.94% for CuHCF. These results suggest that structural water influences the competition between OER and CER, although chlorine evolution remains significant under these chloride-rich conditions. Therefore, the study provides useful insight into the role of hydration in controlling reaction selectivity in saline media, rather than representing direct electrolysis of natural seawater.

Kalaiyarasan et al. [113] reported an efficient urea electrolysis system based on NiHCF nanocubes and NiS nanoframes directly grown on nickel foam. In this configuration, NiHCF/NF was mainly active toward the urea oxidation reaction (UOR), whereas NiS/NF provided the catalytic sites for the HER. NiHCF reached 100 mA cm^−2^ at approximately 1.39 V vs. RHE for UOR, while NiS/NF required an overpotential of 46 mV at 10 mA cm^−2^ for HER. When coupled in a two-electrode NiHCF/NF and NiS/NF electrolyzer, the system achieved 100 mA cm^−2^ at a cell voltage of 1.8 V. Although these experiments were not performed in seawater, the study illustrates how PBA-based electrodes can be integrated with a complementary catalytic phase to reduce the energy demand of hydrogen production.

Zhang et al. [114] presented a self-supporting, highly porous NiFe-HCF electrocatalyst with a hierarchically hollow nanostructure, which exhibits significant catalytic performance toward oxygen evolution in alkaline seawater electrolytes. The electrolyzers consisting of NiFe-PBA and NiMoN achieved a high current density of 500 mA cm^−2^ at a low cell voltage of 1.782 V for the general electrolysis of alkaline seawater, demonstrating excellent durability for 100 h.

Despite these advances, several limitations remain. Most studies report NiHCF performance under simplified or modified conditions, such as synthetic seawater or the addition of alkaline electrolytes, which can significantly alter reaction pathways and suppress competing processes such as CER. As a result, the extrapolation of these findings to real seawater systems remains non-trivial. Furthermore, metrics such as overpotential and current density are often reported without standardized conditions, hindering direct comparison across studies. Taken together, these observations indicate that while NiHCF demonstrates promising stability and selectivity, its practical implementation in seawater electrolysis requires further validation under realistic, additive-free conditions and at industrially relevant current densities.

### 6.2. CoHCF

CoHCF has emerged as a highly active PBA system in saline electrochemical environments, particularly for reactions involving rapid charge transfer and surface redox processes. CoHCF typically exhibits enhanced electronic conductivity and more favourable reaction kinetics, which can be attributed to the electronic configuration of cobalt centres and their interaction within the Fe–CN–Co framework. This often translates into lower overpotentials and higher current densities in both HER and OER processes. However, this increase in activity is accompanied by a more complex interplay with competing reactions in chloride-rich media, requiring careful evaluation of selectivity under realistic conditions [50,115].

In seawater electrolysis, CoHCF-based systems frequently demonstrate improved catalytic performance relative to NiHCF, particularly in terms of current density and kinetic response. Nevertheless, this enhanced activity can also lead to increased susceptibility to the CER, especially under anodic polarization. Experimental evidence indicates that CoHCF may exhibit partial selectivity toward chlorine production in natural seawater, with faradaic efficiencies for CER reaching significant values under certain operating conditions. This behaviour suggests that, while CoHCF can effectively accelerate electrochemical reactions, it may also promote undesired pathways due to less selective adsorption of chloride species compared to more structurally constrained systems such as NiHCF [89,115].

The higher intrinsic reactivity of cobalt-based systems may accelerate this transformation and modify surface chemistry more dynamically under polarization. In saline environments, this can lead to a complex balance between catalytic activation and degradation, as reconstructed surfaces may become more active but also more exposed to chloride-induced corrosion or structural variability. Therefore, the performance of CoHCF in seawater electrolysis must be understood as the result of competing effects between enhanced kinetics and reduced selectivity and durability.

**Figure 16:**
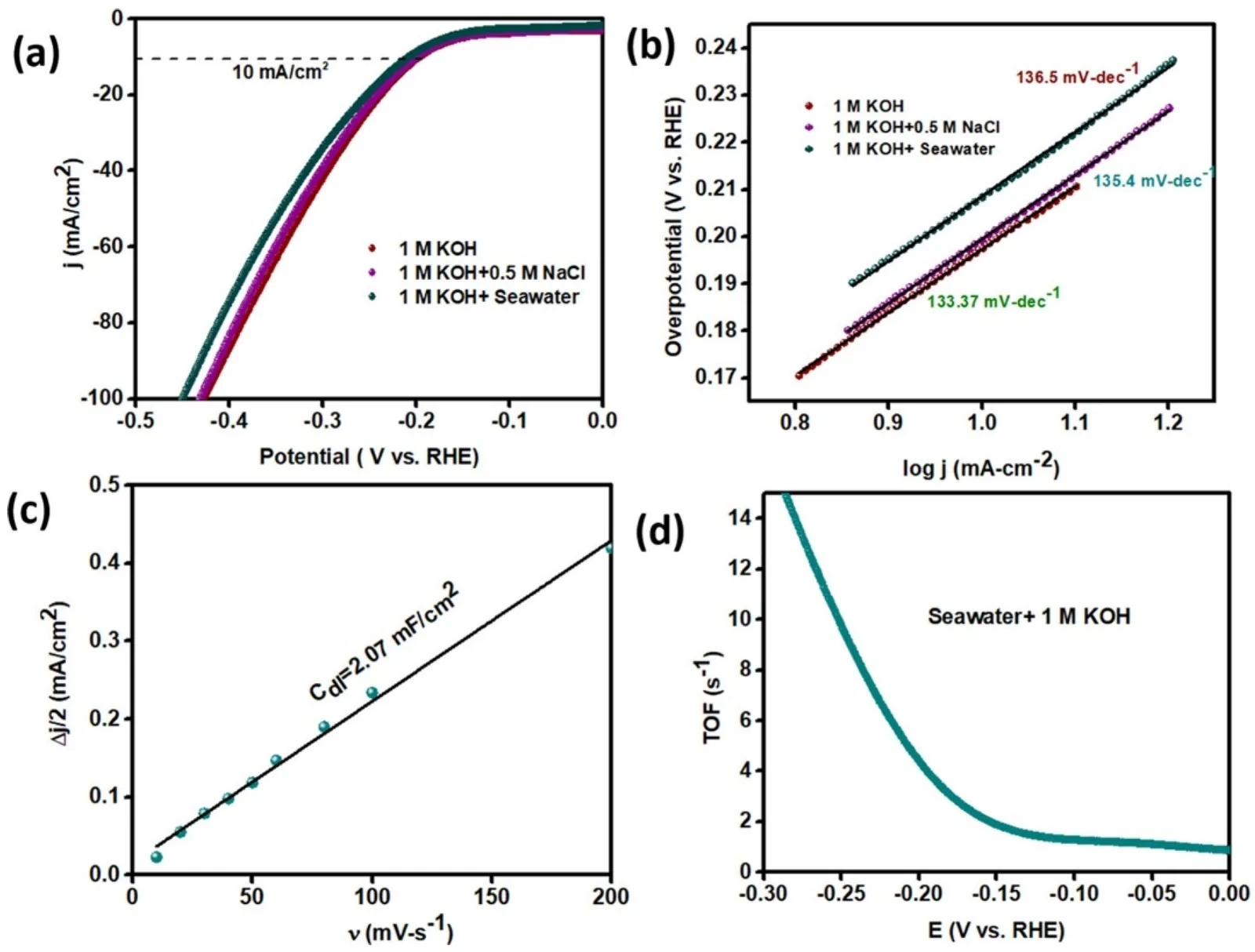
(**a**) HER polarization plots in different media, (**b**) Tafel plot in natural seawater, (**c**) capacitive current vs. scan rate plot, and (**d**) TOF plot in natural seawater of Ni/NCT. Based on Ghosh et al. (2023) [116].

Ghosh et al. [116] developed a two-electrode system for seawater electrolysis combining a CoFePBA-based anode supported on nitrogen-doped carbon nanotubes (CoFePBA/NCNT) with a Ni/NCT cathode. The two electrodes showed complementary catalytic activity: Ni/NCT required an overpotential of 105 mV at 10 mA cm^−2^ for HER, with a Tafel slope of 57 mV dec^−1^, whereas CoFePBA/NCNT reached the same current density for OER at an overpotential of 260 mV and a Tafel slope of 61 mV dec^−1^. When assembled as a complete electrolyzer, the system delivered 10 mA cm^−2^ at a cell voltage of 1.71 V and maintained stable operation for 50 h under saline conditions (Figure 16). The authors associated the OER performance of the PBA-based anode with its interconnected nanosheet morphology, which provides a high density of accessible active sites and facilitates electrolyte penetration and charge transfer.

Studies consistently highlight the rapid charge transfer and high electroactive surface area of CoHCF, which are directly linked to its favourable kinetic response in electrochemical environments. In the context of seawater electrolysis, such characteristics suggest that CoHCF can sustain high reaction rates and accommodate dynamic changes in the electrolyte composition. However, the same features that facilitate fast ion transport and redox activity may also reduce control over surface selectivity, particularly in the presence of competing species such as Cl^−^.

Morphological engineering, such as the development of nanosheet or thin-film architectures, has been shown to further enhance the electrochemical performance of CoHCF by increasing the density of exposed active sites and improving electrolyte accessibility. These nanostructured systems often exhibit lower overpotentials and higher current densities, reinforcing the intrinsic kinetic advantage of cobalt-based PBAs. Nevertheless, the increased surface area and exposure can also amplify interactions with aggressive ionic species, potentially accelerating degradation processes or promoting undesired reactions such as CER, respectively. This highlights the importance of considering not only activity gains, but also the stability and selectivity implications associated with nanoscale design in saline environments.

A critical limitation in the actual CoHCF studies is the insufficient evaluation of long-term stability and selectivity under realistic seawater conditions, while short-term electrochemical performance is frequently reported as promising; fewer studies address sustained operation in the presence of multivalent ions, fluctuating pH, and continuous gas evolution. Moreover, discrepancies in electrolyte composition and testing protocols hinder direct comparison between reported results. Consequently, although CoHCF stands out as a highly active PBA system, its practical applicability in seawater electrolysis remains constrained by challenges related to selectivity control and durability. Addressing these limitations will require a more systematic approach to material design and evaluation, particularly focusing on stabilizing active phases while suppressing chloride-driven side reactions.

### 6.3. CuHCF

CuHCF occupies a distinct position within the family of PBAs, as its primary strengths are associated with ion intercalation, redox reversibility, and selective ion transport rather than intrinsic electrocatalytic activity. In the context of seawater electrolysis, the direct catalytic performance of CuHCF toward HER remains less clearly established than that of Ni- or Co-based PBAs, with most available studies focusing instead on its redox reversibility, ion intercalation, and transport behaviour in saline media. However, its relevance lies in its ability to operate under saline conditions while maintaining high faradaic efficiency and structural reversibility. Importantly, CuHCF has demonstrated stability in natural and synthetic seawater electrolytes, although it remains more susceptible to structural modifications or partial dissolution under prolonged polarization. These observations suggest that CuHCF is not ideally suited as a primary electrocatalyst, but rather as a complementary material within multifunctional electrochemical systems.

In seawater electrolysis systems, this property may be leveraged to regulate the local electrolyte composition, mitigate surface poisoning, or recover valuable ions as part of a broader resource valorization strategy. Therefore, CuHCF should be viewed not as a high-performance electrocatalyst, but as a functional component that complements catalytic processes by enabling ion management and enhancing system-level performance.

Sciacca et al. [117] compared electrodeposited films of NiHCF and CuHCF as rare earth element hosts by ion insertion. CuHCF showed good reversibility and recoverability, with the insertion of heavy elements being more efficient compared to light ones.

**Figure 17:**
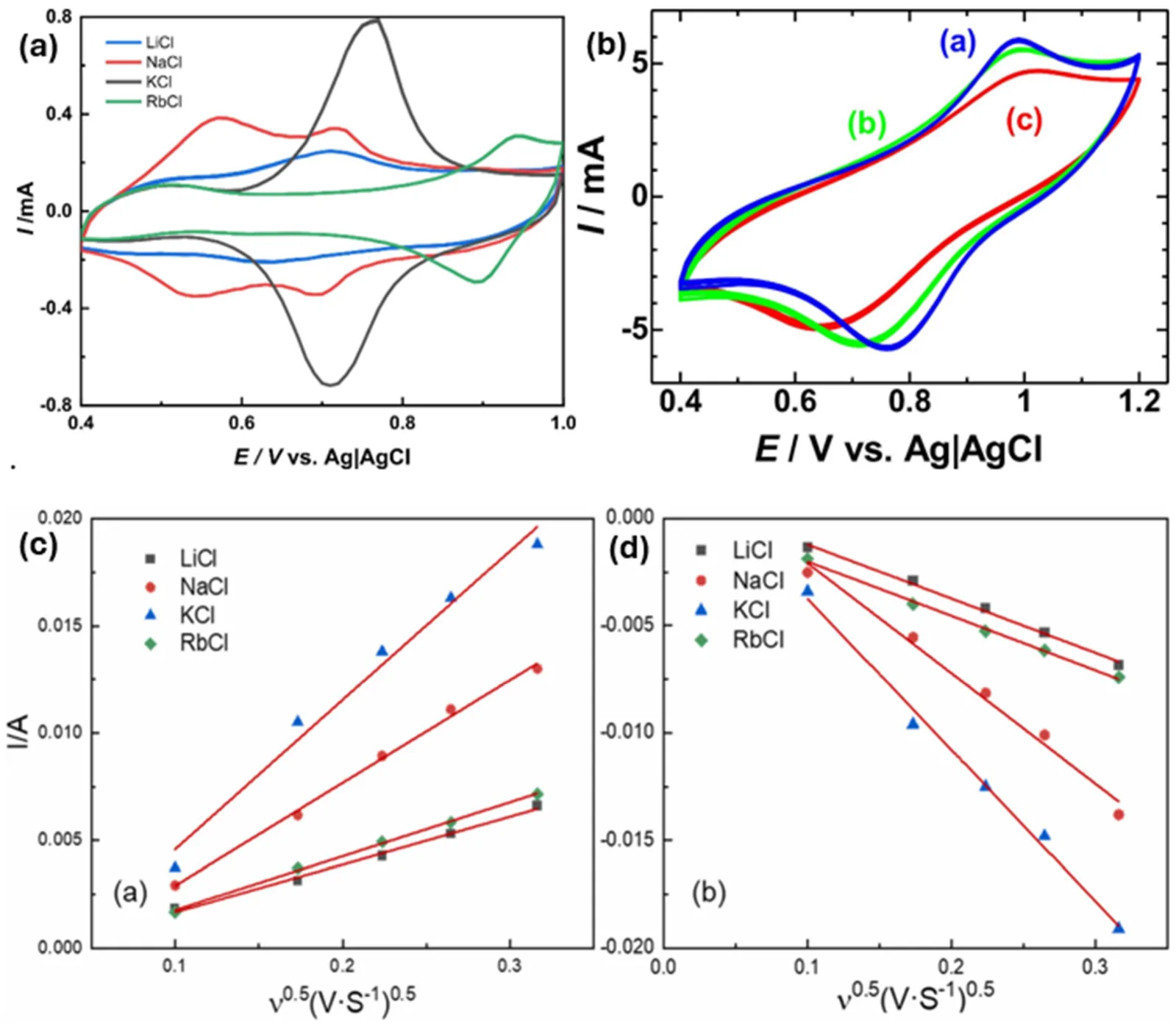
(**a**) Cyclic voltammetry curves of CuHCF in different cationic solutions at the scan rate of 1 mV s^−1^. (**b**) Cyclic voltammetry curves of CuHCF electrode sheet in 1 M RbCl solution at scan rates of (a) 100 mV s^−1^, (b) 50 mV s^−1^, and (c) 10 mV s^−1^. The relationship of ip and v for the alkali metal cations (**c**) reduction and (**d**) oxidation peaks. Based on Zeng et al. (2024) [23].

Zeng et al. [23] investigated the electrochemical behaviour of CuHCF in chloride-rich solutions containing Li^+^, Na^+^, K^+^ and Rb^+^, using cyclic voltammetry to evaluate cation insertion and de-insertion processes. At a scan rate of 1 mV s^−1^, the CuHCF electrode showed distinct redox responses depending on the alkali-metal ion, while experiments performed in 1 M RbCl at 10, 50 and 100 mV s^−1^ confirmed the strong dependence of the peak current on scan rate (Figure 17). The authors concluded that the insertion/de-insertion mechanism is predominantly diffusion-controlled, with K^+^ exhibiting the highest diffusion rate and Li^+^ the lowest, whereas reversible electrochemical behaviour was observed for Na^+^, K^+^ and Rb^+^. These results highlight the ability of CuHCF to regulate ion transport in concentrated saline media, supporting its potential use in selective ion recovery and electrolyte management rather than as direct evidence of HER electrocatalytic activity.

Shuaib et al. [118] incorporated cobalt oxide (Co_3_O_4_) into the CuHCF structure to improve its electrochemical performance. The Co_3_O_4_–CuHCF exhibited a high specific capacitance of 1456 Fg^−1^ at a current density of 2 Ag^−1^, demonstrating a combined contribution from electrical double-layer processes and Faradaic reactions associated with the system’s redox centres. The incorporation of Co_3_O_4_ enhances charge transfer and electronic conductivity.

CuHCF represents a fundamentally different paradigm within PBA-based systems for seawater electrolysis, where its primary contribution lies not in maximizing catalytic activity, but in enabling controlled ion transport and selective electrolyte interactions. While its intrinsic HER/OER performance remains limited, its high redox reversibility, ion selectivity, and operational stability in saline media make it a valuable component in integrated electrochemical platforms. In this sense, CuHCF is best understood as a functional material that complements electrocatalysis by regulating the local chemical environment, mitigating deactivation pathways, and supporting hybrid strategies that couple hydrogen production with ion separation and resource recovery.

### 6.4. MoHCF

Dong et al. [119] studied thin films of molybdenum hexacyanoferrate (MoHCF) deposited on Pt electrodes, showing that their electrochemical response strongly depends on the cation present in the electrolyte. Among the ions evaluated, NH_4_^+^ produced the lowest charge loss, approximately 11%, whereas Ca^2+^ resulted in a considerably higher loss of about 54%, reflecting marked differences in cation transport through the MoHCF framework. Rather than demonstrating direct electrocatalytic activity toward HER or OER, these results highlight the ion-permeable nature of MoHCF and the influence of cation charge and transport on its redox response. This behaviour is relevant when considering PBAs in complex saline electrolytes, where multivalent species such as Ca^2+^ can strongly affect ion exchange and charge-transfer processes at the electrode–electrolyte interface.

## 7. Other PBAs

Beyond Ni-, Co-, and Cu-based systems, a broader range of PBAs incorporating transition metals such as Mn, Fe, Mo, and Bi has been explored in saline electrochemical environments, revealing diverse functionalities and performance profiles. These materials often exhibit distinct electrochemical behaviours depending on their metal centres, coordination environment, and structural features, highlighting the compositional versatility of PBAs as a platform for material design. In many cases, these alternative PBAs do not outperform benchmark catalysts in terms of intrinsic activity; however, they provide valuable insights into how electronic structure, redox chemistry, and ion transport can be tuned to address the specific challenges of seawater electrolysis.

A recurring trend among these systems is the emergence of hybrid or heterostructured materials, where PBAs are combined with metal oxides, phosphides, or conductive carbon phases to enhance performance. Such strategies aim to overcome intrinsic limitations of PBAs, particularly their low electronic conductivity, while leveraging their structural and redox advantages. For instance, the incorporation of secondary catalytic phases can significantly improve HER/OER kinetics, whereas the PBA framework contributes to structural stability and ion management under saline conditions. This synergistic approach reflects a broader shift in the field, where PBAs are increasingly used as precursors, scaffolds, or functional components within composite systems rather than as standalone catalysts.

Despite these advances, the study of alternative PBAs in seawater electrolysis remains fragmented, with limited systematic comparison across materials and operating conditions. Variations in electrolyte composition, pH, applied potential, and testing protocols make it difficult to establish clear performance benchmarks or identify universal design principles. Nevertheless, the diversity of reported systems underscores a key insight: the strength of PBAs lies not in maximizing a single performance metric, but in their ability to integrate multiple functionalities—catalysis, ion transport, and structural adaptability—within a single material platform. Moving forward, the development of next-generation PBAs will likely rely on rational compositional design, controlled defect engineering, and hybridization strategies tailored to the complexities of real saline environments. Zhang et al. [16] synthesized heterostructured trimetallic nanocages based on Ni-CoHCF and Fe-CoHCF using an iterative coprecipitation strategy. The developed electrocatalyst (Ni-Co@Fe-CoPBA) exhibits remarkable HER electrocatalytic performance with small overpotentials of 43 and 183 mV to drive a current density of 10 mA cm^−2^ in alkaline freshwater and simulated seawater, respectively. The fabricated two-electrode electrolyzer reportedly achieves high output current densities of 44 and 30 mA cm^−2^ at a low potential of 1.6 V in alkaline freshwater and simulated seawater, respectively, exhibiting remarkable structural stability during a 100 h test.

Wang et al. [120] reported the synthesis of dehydrated BiHCF as a highly active cathode material characterized by a 3e^−^ transfer mechanism (Bi^3+^/Bi^0^), resulting in a higher charge density compared to conventional 2e^−^ PBAs. Cyclic voltammetry analysis showed well-defined redox pairs of 1.6–1.9 V (reduction) and 3.0–3.3 V (oxidation), demonstrating high electrochemical reversibility and efficient charge transfer kinetics. Furthermore, the dehydrated material reduces crystal defects caused by interstitial water and improves electronic conductivity, resulting in a lower charge transfer resistance (16.7 Ω) compared to hydrated BiHCF. The CoHCF, in turn, stands out for its higher catalytic activity and faradaic efficiency in photoelectrochemical and direct electrolysis configurations. CoFe–PB coatings on BiVO_4_ achieve significant enhancements, with oxygen faradaic efficiencies exceeding 95%. Nonetheless, during direct electrolysis of natural seawater, CoHCF exhibits a faradaic efficiency for the Cl^−^ evolution reaction of up to 33%, indicating that although the material is structurally stable, OER/CER selectivity remains a critical challenge.

A critical limitation across the literature is the inconsistency in experimental protocols, which complicates direct comparison between studies and obscures the identification of clear performance trends. Variations in parameters such as electrolyte composition (synthetic vs. natural seawater), pH adjustment, presence of additives (e.g., KOH), and current density ranges can lead to significant discrepancies in reported results. Moreover, the frequent use of short-term measurements fails to capture degradation processes that become dominant under extended operation. To advance the field, there is a clear need for standardized testing methodologies that incorporate realistic saline conditions, long-term stability assessments, and comprehensive reporting of electrochemical parameters, enabling more reliable benchmarking and rational material design. In catalytic applications, NiHCF exhibits an OER overpotential of approximately 320 mV at 10 mA cm^−2^, maintaining stability for more than 100 h under alkaline conditions (synthetic seawater + 1 M KOH), demonstrating its resistance to degradation under prolonged polarization. However, reaction selectivity remains a major challenge under natural seawater conditions. In this regard, Hegner et al. [115] reported that CoHCF, although electrochemically active and structurally stable in natural seawater (pH ~8, ~0.55 M Cl^−^), exhibited a Faradaic efficiency for chlorine evolution exceeding 30%. This result clearly demonstrates that high catalytic activity and structural stability do not necessarily translate into selective OER performance when chloride is present at seawater concentrations.

In terms of electrocatalytic stability, Ni- and Co-based PBAs have demonstrated considerable performance in saline environments through continuous operation. Specifically, NiHCF and CoHCF maintain their activity for periods between 50 and 100 h, with a decrease in current density of less than 10%, a low and acceptable loss that suggests some resistance to structural degradation and surface passivation during HER. In contrast, CuHCF exhibits greater instability due to partial dissolution or structural reconfiguration under highly polarized conditions. It is worth mentioning that, due to the scarcity of electrocatalytic research in saline environments, the results presented in this manuscript serve as a contextual reference, linking energy storage applications with catalytic activity. They directly reflect demanding operational conditions, such as cyclic stability, in electrocatalytic systems in seawater. A critical issue emerging from the analysis is the lack of standardization in electrochemical metrics. Potentials are reported against different reference electrodes (Ag/AgCl, RHE, NHE, K^+^/K, Al^3+^/Al), and current densities are expressed in both mA cm^−2^ and mA g^−1^, hindering direct quantitative comparison across studies. Furthermore, many investigations are conducted in synthetic electrolytes or with additives (e.g., KOH, Na_2_SO_3_), limiting their representativeness with respect to real seawater.

In this context, reliable benchmarking of PBA-based materials demands standardized methodologies that reflect the true operating conditions of seawater electrolysis. This includes the use of additive-free electrolytes, consistent reference electrode calibration, and extended durability testing at industrially relevant current densities. Furthermore, the integration of advanced characterization techniques, particularly in situ and operando approaches, will be critical to elucidate the dynamic transformations occurring at the electrode–electrolyte interface.

Ultimately, advancing the field will depend not only on improving material performance, but also on establishing robust evaluation protocols that enable meaningful comparison across studies. Such an approach will facilitate the identification of genuine structure–property–performance relationships and support the rational design of next-generation PBAs tailored for efficient and stable operation in complex saline environments.

Baghendra Singh et al. [121] reported the synthesis of multimetallic PBA nanoparticles composed of MnFeCoNiCu-PBA, prepared through a simple room-temperature route and evaluated as anodic electrocatalysts for the OER and benzyl alcohol oxidation (BAO). The synthesis involves the direct mixing of aqueous solutions containing Mn^2+^, Co^2+^, Ni^2+^, and Cu^2+^ with potassium ferricyanide (K_3_[Fe(CN)_6_]). For the OER, the MnFeCoNiCu-PBA material reached a current density of 100 mA cm^−2^ at 1.67 V vs. RHE, together with favourable reaction kinetics and reduced charge-transfer resistance. Under anodic polarization, the PBA undergoes electrochemical reconstruction toward metal hydroxide/oxyhydroxide species (M(O)OH), which constitute the active surface during oxidation. An additional feature of this work is the use of BAO as an alternative anodic reaction to OER, reducing the energy demand of the overall electrochemical process and thereby favouring H_2_ production at the cathode while simultaneously enabling chemical valorisation. In contrast, Fei Ma et al. [92] propose a more targeted and robust strategy based on plasma-induced generation of [Fe(CN)_6_] vacancies in a trimetallic NiCoFe-based PBA. This approach directly modifies the chemical environment of the metal centres, creating undercoordinated active sites with enhanced affinity for reaction intermediates. The introduction of vacancies promotes the transformation of the material into NiCoFeOOH-type phases, which are responsible for the observed catalytic activity. As a result, the system achieves an overpotential of ~293 mV at 100 mA cm^−2^ in seawater, significantly lower than the ~405 mV required for the unmodified material, while maintaining stability beyond 110 h in highly saline electrolytes. Furthermore, the retention of Fe and the formation of NiCoFeOOH favour OH^−^ adsorption over Cl^−^, thereby improving both selectivity and corrosion resistance. Nevertheless, despite its robustness under realistic conditions, its scope remains somewhat limited, as it focuses exclusively on the OER.

Baghendra Singh et al. [121] are distinguished by their impact on energy efficiency and chemical valorisation, whereas Fei Ma et al. [92] provide deeper insight and more realistic performance under demanding operating conditions, highlighting that progress in the field requires both functional innovation and precise structural control, alongside long-term stability.

Hualin Jiang et al. [122] reported that a CoFe-PBA/NiFe-LDH material was synthesized on Ni foam. This system achieves competitive bifunctional performance in alkaline electrolysis, with η_10_ ≈ 79 mV for the HER, 212 mV for the OER, and 1.48 V for overall water splitting, along with high operational stability (>100 h). These results demonstrate an effective improvement in kinetics and charge transfer, primarily associated with the formation of an intimate heterostructure, in which the PBA acts as a porous framework facilitating ionic transport. At the same time, the interface between the PBA and LDH phases promotes electronic transfer by reducing charge-transfer resistance and enabling more efficient activation of the Ni/Fe metal centres during the reaction.

Nevertheless, the information provided regarding the use of a well-defined heterostructure allows for a clearer isolation of interfacial effects on catalytic activity, contributing to a more consistent interpretation of the results. In this sense, it represents a methodologically sound and reproducible study, albeit without significant emphasis on operational challenges such as electrolysis in saline media.

Nahyun Lee et al. [123] report a hybrid material composed of CoCuFe-PBA@P-MoS_2_, consisting of a trimetallic PBA dispersed on a phosphorus-doped semiconductor support. Unlike high-entropy PBAs such as MnFeCoNiCu, this system does not rely on increased compositional complexity; instead, in contrast to NiCoFe-PBA materials, it adopts a strategy based on metal–support interactions. Phosphorus-doped MoS_2_ plays a crucial role by modifying the electronic structure, generating defects and redistributing charge density, thereby enhancing conductivity and optimizing the adsorption energetics of OER intermediates. In terms of performance, the material exhibits notable OER activity, with an overpotential of 186 mV at 10 mA cm^−2^, a Tafel slope of 68.9 mV dec^−1^, and a Faradaic efficiency of ~97.6%. However, it does not match the versatility reported by Baghendra Singh et al. [121] nor the robustness under realistic conditions demonstrated by Fei Ma et al. [92].

Tao Pan et al. [124] report the synthesis of a MnCoFe PBA-P compound with a hollow cubic structure, whose primary advantage lies in its influence on transport phenomena. The hollow cubic architecture increases the electrochemically active surface area and alleviates diffusional limitations, facilitating OH^−^ access to active sites and promoting the efficient release of O_2_ bubbles during the reaction. This mitigates surface blockage and enhances the effective utilization of the material under operating conditions. In this case, the OER performance is moderate, with an overpotential of 327 mV at 10 mA cm^−2^ and a Tafel slope of 92.8 mV dec^−1^, remaining below that of the best-performing systems discussed previously, although it exhibits good stability (~40 h). In this regard, the synthesis strategy leads to improvements primarily associated with structural architecture, optimizing mass transport and operational stability during electrocatalysis, but without providing significant electronic enhancements.

Xuechun Zhou et al. [125] report a multimetallic FeCoNiCr_6_P material exhibiting clearly superior performance, achieving an overpotential of 268.3 mV at 100 mA cm^−2^ for the OER, a total cell voltage of 1.58 V for overall water splitting, a Faradaic efficiency close to 100%, and good stability. This behaviour is primarily attributed to the role of Cr in modulating the electronic structure of the active centres, where its incorporation enables the tuning of the adsorption energy of OER intermediates and the stabilization of high oxidation states in Ni and Co. The presence of multiple metals promotes a more efficient redistribution of charge, enhancing electron-transfer kinetics and explaining its superior performance compared to previously reported systems. Yu-Ru Liu et al. [91] introduce a relevant contribution by focusing specifically on the HER, a reaction that has been less explored in the PBA literature compared to the OER, where performance is often limited by low conductivity and suboptimal hydrogen adsorption. The approach proposed by the authors highlights how modifications in the electronic structure and charge transport can directly impact the kinetics of H_2_ formation, beyond the dominant reconstruction mechanisms typically observed in OER. Through their synthesis methodology, which incorporates carbon nanotubes (CNTs) to form a conductive network that reduces electronic resistance and facilitates charge transfer, the system can operate at high current densities. These characteristics account for an overpotential of 198 mV at 100 mA cm^−2^ obtained in 1.0 M KOH and sustained stability at 0.5 A cm^−2^ for 48 h, demonstrating a clear improvement compared to reference bimetallic systems.

This comparison further supports the need to redefine performance criteria in seawater electrolysis. Rather than focusing exclusively on activity benchmarks derived from ideal conditions, materials such as PBAs highlight the importance of stability, selectivity, and environmental compatibility. This shift in evaluation criteria represents a key conceptual contribution of this work, emphasizing that the suitability of a material must be assessed within the context of its operating environment.

A comparative analysis of PBAs with state-of-the-art catalysts (Table 1) reveals that, despite their structural versatility and stability in saline environments, PBAs still exhibit significantly higher overpotentials and slower kinetics, particularly for the HER.

This discrepancy highlights a fundamental limitation associated with the intrinsic electronic conductivity of PBAs, where lattice-mediated charge transport restricts reaction rates. In contrast, metallic systems provide efficient electron pathways, enabling lower activation barriers and faster kinetics.

For anodic processes, PBA-derived systems show improved performance due to surface reconstruction toward (oxy)hydroxide phases; however, their selectivity under chloride-rich conditions remains a critical challenge, particularly due to the competition between OER and CER. These observations suggest that PBAs may be better understood as dynamic precursors rather than stable catalytic phases, aligning with recent trends in electrocatalysis where active sites are generated in situ.

The compilation of these studies demonstrates that the performance of PBA-based materials in saline media cannot be compared using only overpotential or current density. Significant variations exist due to differences in electrolyte composition, chloride concentration, pH, and consequently, the test conditions can significantly affect activity, stability, and OER/CER selectivity. Therefore, it is important to distinguish between natural seawater, simulated seawater, alkaline seawater, and simplified chloride electrolytes. Table 1 presents the PBA-based systems evaluated under these conditions, including their electrochemical performance, stability, and reaction selectivity.

Beyond the individual performance metrics reported in Table 1, the comparison reveals that the role of PBAs in seawater electrolysis cannot be described solely in terms of catalytic activity. Their behaviour is governed by the simultaneous interaction of three factors: the ability to sustain charge transfer and promote the desired electrochemical reaction, the interaction of the porous framework with ions present in the electrolyte, and the structural response of the material during prolonged polarization. These functions are closely interconnected. Changes in composition, vacancy concentration, hydration, or surface chemistry may improve the accessibility of active sites and ion transport, but can also modify chloride adsorption, structural stability, or the tendency of the material to reconstruct under operating conditions. Therefore, the performance of a PBA in seawater should be interpreted as the result of a balance between activity, ion–electrolyte interactions, selectivity, and structural evolution, rather than through a single descriptor such as overpotential.

Within this framework, the comparison between the different systems in Table 1 shows that composition alone does not determine their electrochemical response. The function of the PBA within the electrode and, particularly, the characteristics of the saline electrolyte strongly influence the observed performance.

The results shown at the top of Table 1, related to simpler PBAs, demonstrate that although these materials can maintain activity in the presence of chlorides, selectivity remains a significant limitation. In the research by Hegner et al. [115] on CoHCF, water oxidation occurs simultaneously with Cl^−^ oxidation, demonstrating direct competition between OER and CER. A similar situation is observed in the work of Raja et al. [112] in the comparison between NiHCF and CuHCF, where the selectivities towards OER reach 26.47% for NiHCF-1, 14.56% for NiHCF-2, and 10.94% for CuHCF. These results show that the material’s stability in saline environments does not necessarily imply high electrochemical selectivity and that structural variables, such as the water content of the PBA, can influence this behaviour.

Strategies involving structural modification and the formation of heterostructures allow for a significant improvement in catalytic activity. This strategy was demonstrated by Zhang et al. [16] to determine the HER parameters of Ni–Co@Fe–Co PBA, requiring an overpotential of 183 mV at 10 mA cm^−2^ in simulated seawater, while Wang et al. [129], for a PBA-350 derivative, achieved η_100_ = 159.5 mV under similar conditions. Superior performance was obtained by Wang et al. [128] with Ni-MoS_x_@NiMnFe-PBA, which exhibits overpotentials of 71 mV for HER and 260 mV for OER at 10 mA cm^−2^, also reaching a cell voltage of 1.513 V for overall electrolysis. These results suggest that the contribution of PBA is not necessarily limited to acting as the main catalytic phase. In several systems, its structure also functions as a support, protective layer, or medium to modify charge transport and the interaction between the catalyst and the electrolyte.

This trend is also observed in materials intended for OER. The work carried out by Ma et al. [92] on the controlled generation of vacancies in N_2_–NiCoFe-PBA resulted in a decrease in overpotential from 405 to 293 mV in simulated seawater and from 434 to 323 mV in natural seawater. Regarding the research carried out by Shi et al. [127] on Ni(OH)_2_/NiFe-PBA, they reported OER values of 292 mV. However, in these cases, it is important to consider that the original PBA can be modified during polarization. The investigation revealed the formation of phases such as NiOOH, Ni(Fe)OOH, and NiCo-FeOOH, indicating that some of the observed activity is associated with surface reconstruction processes. Therefore, some PBAs should be better understood as precursors or structures capable of promoting the formation and stabilization of the catalytically active phase during operation.

Regarding stability under saline conditions, significant progress has also been made. While some of the earlier systems were evaluated for periods of approximately 50–100 h, more recent materials achieve considerably longer operating times. The work of He et al. [107] with NiHCF achieved stable performance for approximately 1000 h at 1 A cm^−2^, while Shi et al. [127] were able to use Ni(OH)_2_/NiFe-PBA for 1000 h at 500 mA cm^−2^, and Ge et al. [130] with R-MnFeO_x_/PBA, stability exceeded 1150 h at 1000 mA cm^−2^. Also noteworthy is the report by Wang et al. [128] with NiMoS_x_@NiMnFe-PBA, which showed stability of up to 2500 h at 500 mA cm^−2^. In these studies, they demonstrate improved durability associated with strategies aimed at limiting Cl^−^ interaction with the surface, whether through protective layers, surface reconstruction, carbonaceous structures, or chloride repulsion effects.

In general, the comparison in Table 1 shows that the advancement of PBAs for seawater electrolysis cannot be evaluated solely based on overpotential. Stability, selectivity against CER, and the ability to resist corrosion or salt accumulation are equally relevant. At the same time, it should be considered that a significant portion of the best results have been obtained in simulated seawater, alkaline media, or simplified NaCl solutions. Studies conducted directly in natural seawater remain less numerous, making it difficult to establish a completely homogeneous comparison between materials and to clearly define which systems are best suited for real-world operating conditions.

## 8. Insights from Other Electrochemical Applications of PBAs

Although some of the applications discussed in this section are not directly related to seawater splitting, they provide useful information on key properties of PBAs, such as ion transport, redox reversibility, electronic conductivity, and structural stability. These characteristics are also important in seawater electrolysis, where charge transfer, ion interactions, and structural changes during operation can strongly affect catalytic performance. Therefore, these studies are considered not as direct benchmarks for seawater electrolysis, but as complementary evidence for identifying structure–property relationships relevant to the design of PBA-based electrodes for chloride-rich environments.

The comparative analysis presented in Table 2 shows that the electrochemical behaviour of PBAs cannot be described by a single performance metric, but rather depends on the interplay between structure, composition, and application. NiHCF illustrates this versatility particularly well. In electrosorption and desalination systems, it exhibits moderate capacities of 43–55 mAh g^−1^ together with good cycling stability (>100 cycles), highlighting the structural robustness of its framework under saline conditions [134]. When combined with conductive materials such as rGO or modified through K^+^ incorporation, capacities of up to 138 mAh g^−1^ and energy densities of up to 58.7 Wh kg^−1^ have been reported [135,136]. These improvements indicate that electronic conductivity and electrode architecture strongly influence the electrochemical response of NiHCF. In electro-mining applications, its high Cs^+^ removal efficiency (99.4%) further demonstrates the ion-selective character of the PBA framework [137].

CoHCF-based systems show a different electrochemical profile. In energy-storage applications, CoHCF exhibits competitive performance, with reported capacities of approximately 110 mAh g^−1^, although its cycling behaviour can depend strongly on electrode composition and conductive additives [138]. In electrocatalytic systems, reaction selectivity becomes particularly important: during direct electrolysis of natural seawater, Faradaic efficiencies for chlorine evolution of up to 33% have been reported, indicating substantial competition between the OER and CER [115]. By contrast, when CoFe-PB is incorporated as a surface layer on photoelectrodes such as BiVO_4_, high oxygen Faradaic efficiencies and improved photoelectrochemical performance have been achieved [81]. In these systems, the PBA layer contributes to interfacial charge transfer and charge separation, showing that its function can vary considerably depending on the electrode configuration.

CuHCF shows a different electrochemical response from Ni- and Co-based PBAs. In battery deionization systems, relatively low capacities of approximately 24.7 mAh g^−1^ and progressive degradation after extended cycling have been reported, indicating limitations in charge-storage performance under these conditions [139]. However, electrochemical studies of CuHCF in chloride-containing electrolytes show highly reversible redox behaviour and diffusion-controlled cation insertion and de-insertion. This response reflects the ability of its open framework to accommodate and transport different ionic species while maintaining reversible redox activity [23]. Consequently, CuHCF has attracted particular interest in ion separation, selective ion recovery, and electrochemical desalination systems, where ion-exchange behaviour is more important than high charge storage capacity.

The inclusion of KMnHCF highlights the effect of combining multiple redox-active metal centres with conductive additives [140]. KMnHCF reaches capacities of up to 119 mAh g^−1^ and an energy density of approximately 464 Wh kg^−1^, outperforming NiHCF and CoHCF under the reported conditions. This improvement is mainly associated with the redox contribution of Mn and the enhanced electronic conductivity provided by CNTs, which also helps limit metal dissolution and preserve the PBA framework during cycling. Nevertheless, the reported capacity retention of approximately 91% after 100 cycles indicates that structural degradation remains a relevant limitation.

In NaM-nHCF systems evaluated for Al-ion batteries, moderate capacities of approximately 50–60 mAh g^−1^ are obtained together with good coulombic stability [141]. The electrochemical response is strongly influenced by the multivalent nature of the charge carrier, since the higher ionic charge restricts diffusion within the PBA framework. The best performance is observed at intermediate electrolyte concentrations, around 2 M, indicating that ion mobility and electrolyte concentration must be balanced to maintain efficient transport through the structure. These results indicate that increasing electrolyte concentration does not necessarily improve the electrochemical response of PBAs, as ion mobility can become increasingly restricted at higher concentrations.

Multimetallic systems such as Na/Co/NiHCF show improved electrochemical performance, reaching capacities of up to 116.4 mAh g^−1^ and energy densities of approximately 128 Wh kg^−1^ [142]. The incorporation of Co and Ni modifies the local electronic environment of the PBA framework and expands its redox activity, contributing to improved charge storage and electrochemical reversibility. These results illustrate how metal substitution can be used to tune the electronic and redox properties of PBAs. However, the reported performance was obtained in controlled electrolytes such as NaSO_3_CF_3_ and NaClO_4_.

**Table 2:** Selected electrochemical applications of PBAs and transferable structure–property relationships relevant to saline electrolysis.

Cathode Material (PBA)	Anode Material	Potential Range [V]	Specific Capacity [mAh g^−1^]	Electrochemical Application	Electrolyte	Electrode Mass [mg]	Current Density [mA/cm^2^]	Energy Density [Wh kg^−1^]	Others Parameters	Ref.
NiHCF (rhombohedral and cubic)	(NaTi_2_(PO_4_)_3_	0.2 to 1.2 (vs. Ag/AgCl)	55 (rhombohedral) and 43 (cubic)	Electrochemical desalination (ESD)	20 mM NaCl and 0.4 M NaCl	0.7–1.0 mg cm^−2^	0.1–1.0	-	Stability > 100 cycles. Active area 1 cm^2^	[134]
NiHCF/rGO reduced graphene	AC	0 to 1.6	-	Asymmetric hybrid supercapacitor	1 M Na_2_SO_4_	1.0 mg	0.5–10	~ 58.7	87.2% retention after 3000 cycles. Coulombic efficiency >98%	[136]
NiHCF (coating on CDI electrode)	AC	−1.2 to 1.2	-	Selective electrochemical removal of cesium (137Cs^+^) from seawater	Synthetic seawater with Cs^+^ at 1 ppm	3–5 mg	±1.0	-	Cs^+^ adsorption efficiency 99.4% in 60 s.	[137]
Na/NiHCF	(NaTi_2_(PO_4_)_3_	1.27	65	Coin cell	1 M Na_2_SO_4_	-	0.65	42.5	>88% retention after 250 cycles at 79 mA g^−1^	[143]
Na/Co/NiHCF	NaTi_2_(PO_4_)_3_	0.8 to 1.8	116.4	Three-electrode system	5 M de NaSO_3_CF_3_	-	50 mA/g	128	>88% retention after 100 cycles at 100 mA g^−1^	[142]
Na/Co/NiHCF	NaTi_2_(PO_4_)_3_	0 to 1.1	85	Three-electrode system	6 M NaClO_4_	-	70 mA/g	121	90% after 100 cycles	[144]
Na/CoHCF	AC	0.3 to 1.0	110.8	Three-electrode system	1 M Na_2_SO_4_	-	-	-	100 cycles. 240 mA g^−1^ (2C): 71.5% (without additive), 100% (with additive) capacity retention	[138]
CoHCF (thin film on CPE)	Pt	−0.2 to +0.6 (vs. Ag/AgCl)	-	Selective adsorption of K^+^ from seawater	Synthetic seawater (13 mM K^+^)	2–3 mg/cm^2^	±0.1–0.5	-	K^+^ adsorption capacity: 6.5 mmol g^−1^	[145]
CoFe-PB (CoHCF) coating	BiVO_4_ (photoanode)	0.3 to 1.23 vs. RHE (onset 0.3 V vs. RHE; shift of −0.8 V compared to bare BiVO_4_)	-	Photoelectrochemical water splitting (OER under solar illumination)	0.1 M potassium phosphate buffer (pH 7) and 1 M Na_2_SO_3_ (hole scavenger, pH 7.9)	-	0.95 at 1.23 V vs. RHE (6× higher than bare BiVO_4_)		Photovoltage increased to 0.60 V; >95% Faradaic efficiency for O_2_; stability > 50 h with <10% decay; band gap of BiVO_4_ unchanged (2.4–2.45 eV)	[81]
CuNiHCF	AC	1	-	Sodium or potassium ion battery	1 M NaNO_3_ or 1 M KNO_3_	5–10 mg/cm^2^	-	-	-	[146]
CuHCF (co-precipitated; on graphite paper)	Pt	CV: Na^+^ 0.70, K^+^ 0.72, Rb^+^ 0.89 (vs. Ag/AgCl); scan 10–100 mV s^−1^	-	Ion intercalation/extraction; voltammetry en simulated brine y 1 M Li^+^/Na^+^/K^+^/Rb^+^	1 M LiCl/NaCl/KCl/RbCl^−^ simulated brine	-	-	-	Diffusion-controlled kinetics. Reversible redox for Na^+^, K^+^, and Rb^+^ (ΔEp < 59 mV). Two redox pairs appear in Na^+^ (Fe^3+^/Fe^2+^ and Cu^2+^/Cu^+^+Fe^3+^/Fe^2+^).	[23]
CuHCF	CuHCF	−0.6 to +0.6 V	24.7 (from 89 full capacity in 1 M NaCl)	Battery Electrode Deionization (BDI) for low-salinity desalination	25–50 mM NaCl (AEM/CEM stacks)	-	0.28 to 1.41	-	Charge efficiency 80%. Cycle stability: 30% charge after 50 cycles at ±0.6 V; improved to 14% at ±0.3 V.	[139]
K_2_MnFe(CN)_6_ (KMnHCF)	K metal	2.7–4.4 vs. K^+^/K	119 (0.1 C), 108 (pristine KMnHCF)	Potassium-ion battery (PIB)	1 M KPF_6_	2.8 to 3.4	-	464	CNT reduces Mn dissolution and improves conductivity; 91% capacity retention after 100 cycles	[140]
NaMnHCF	Treated Al	0.6–1.9 V vs. Al^3+^/Al	50 to 60	Rechargeable aqueous Al-ion battery (AIAB)	2 M Al(OTF)_3_ (optimized); also tested 0.1–5 M	-	30 to 100	130 to 60 over 275 cycles	Stable Coulombic efficiency; performance better in 2 M vs. 5 M (“water-in-salt” not optimal with TAl anode)	[141]

AC: Activated carbon.

Studies from energy-storage systems also provide useful insight into strategies for addressing some intrinsic limitations of PBAs. The incorporation of conductive carbon phases or multiple metal centres can improve charge transport, redox activity, and structural stability during electrochemical cycling. Although these results cannot be directly extrapolated to seawater electrolysis, the same design principles are relevant because limited conductivity and structural degradation also affect PBA-based electrocatalysts. Their effectiveness, however, must be confirmed under chloride-rich electrolytes and electrocatalytic operating conditions.

## 9. Challenges and Opportunities for PBAs in Seawater Electrolysis

This work provides a conceptual shift in the understanding of PBAs in seawater electrolysis by framing them as multifunctional materials rather than conventional electrocatalysts. The analysis demonstrates that their performance is governed by the coupling between structure, ion transport, and electrolyte chemistry, challenging traditional evaluation approaches based solely on catalytic activity. By integrating these aspects, this review establishes a more comprehensive framework for assessing material performance under realistic conditions, highlighting the limitations of current methodologies and the need for more representative evaluation protocols.

As reported by Sterzinger et al. [94], in high-chloride media, the Fe–CN–M ligand network can degrade due to competition with species such as Cl^−^ and OH^−^. In this context, Cl^−^ ions can coordinate with metal centres (Fe or M), forming soluble complexes, while OH^−^ ions promote hydrolysis processes and the formation of metal hydroxides, leading to a progressive structural weakening of the Fe-CN-M bonds. Cl^−^ ions primarily act at the surface or in sites with defects or vacancies, inducing a dissolution process and structural reconfiguration, resulting in a loss of crystalline order and a decrease in the electrocatalytic stability of the material under prolonged operating conditions. Related studies on vacancy-engineered PBAs have also shown that defect chemistry can strongly influence Fe leaching and structural stability during OER [147].

At the anode, CER competes with OER, especially above 1.36 V vs. RHE, generating Cl_2_ and species such as HClO that can oxidize and deactivate the catalytic surface [90,148,149]. This situation is further aggravated in real seawater, where Mg^2+^ and Ca^2+^ are typically present at concentrations of approximately 50–55 mM and 10 mM, respectively. Under alkaline conditions, these ions can precipitate as Mg(OH)_2_ and Ca(OH)_2_, progressively blocking active sites and limiting electrolyte access to the electrode surface [18,104]. Yao et al. [18] point out that, under real seawater conditions, electrolysis faces additional problems: the high concentration of Cl^−^ ions (≈0.5 M) favours the CER, which competes with OER due to its thermodynamic proximity and simpler kinetics, causing accelerated corrosion of the electrodes. At the same time, HER at the cathode locally raises the pH (>9.5), promoting the precipitation of Mg(OH)_2_ and Ca(OH)_2_, which block the active sites and increase the charge transfer resistance.

For these reasons, it is necessary to conduct research and development of materials to improve structural stability under prolonged electrolysis and the competition between desired and undesired reactions. Many PBAs have a conductivity <10^−7^ S cm^−1^ in the pure state, requiring support on conductive materials such as glassy carbon or carbon nanotubes to improve electronic transport due to low electronic conductivity [27,33].

The integration of PBAs with conductive metal oxides (such as RuO_2_, NiFe LDH) or by doping them with metals such as Fe, Cu, or Ru has been shown to synergistically improve catalytic activity. For example, Ghosh et al. [116] showed that the interconnected nanosheet morphology of CoFePBA/NCNT increases the electrochemically accessible surface and facilitates electrolyte transport toward the active sites, contributing to its OER performance. This illustrates how morphological control in PBA-based materials can improve active-site accessibility and ion transport. Goda et al. [43] report strategies such as cyclic voltammetry-assisted electrodeposition or coprecipitation in the presence of surfactants that allow control of crystal size (10–100 nm) and surface roughness, key aspects for increasing catalytic efficiency [43,150].

Future research on PBAs for seawater electrolysis should prioritize rational materials design strategies that explicitly link structure, composition, and electrochemical function. Controlled defect engineering through modulation of [M’(CN)_6_] vacancies, hydration levels, and coordination environments offers a promising pathway to optimize both catalytic activity and ion transport [147]. Similarly, compositional tuning via multimetallic incorporation can be leveraged to adjust electronic structure and adsorption energetics, enabling improved selectivity toward desired reactions. In this regard, the development of compositionally complex or high-entropy PBA systems represents an emerging direction, where synergistic interactions between multiple metal centres may enhance both catalytic performance and resistance to degradation in chloride-rich environments [62,91,125].

Another critical avenue for advancement lies in the design of hybrid and hierarchical architectures that integrate PBAs with conductive and catalytically active phases. Combining PBAs with materials such as carbon nanotubes, graphene, transition metal oxides, or layered double hydroxides can simultaneously address limitations in electronic conductivity and catalytic activity. More importantly, such hybrid systems enable spatial and functional separation of roles within the electrode, where PBAs contribute to ion regulation and structural stability, while secondary phases provide high intrinsic catalytic activity. This approach aligns with the concept of multifunctional electrode design, which is particularly relevant for seawater electrolysis systems operating under complex and dynamic conditions [116,122,128].

Finally, bridging the gap between laboratory-scale studies and real-world implementation remains a fundamental challenge for the field. Future work should emphasize testing under realistic conditions, including the use of natural seawater without additives, operation at industrially relevant current densities, and long-term stability assessments. In parallel, system-level considerations such as reactor design, flow conditions, and integration with desalination or resource recovery processes must be incorporated into material development strategies. Advancing PBAs from promising laboratory materials to viable technological solutions will therefore require a coordinated effort that combines fundamental understanding, materials engineering, and process-level optimization [20,104,149].

## 10. Conclusions

This manuscript establishes an integrated perspective on the use of PBAs in seawater electrolysis, moving beyond the traditional approach of electrolysis in neutral or alkaline media that focuses exclusively on catalytic activity. Unlike previous studies, where PBAs are treated as conventional electrocatalysts, this work demonstrates that their behaviour is governed by a complex interplay between structure, ion transport, and electrolyte chemistry, thereby redefining their role as multifunctional materials in real electrochemical systems. One of the main contributions lies in demonstrating that the performance of PBAs cannot be evaluated solely through conventional metrics such as overpotential or current density, but must instead be analyzed in terms of selectivity, stability, and tolerance to aggressive species such as Cl^−^ present in seawater. Although PBAs generally exhibit lower intrinsic activity than noble-metal catalysts, several PBA-based systems have shown good operational stability and the ability to retain electrochemical functionality in highly saline environments, where chloride-induced corrosion and competing reactions can significantly affect catalyst performance.

Furthermore, this manuscript introduces a critical perspective on the dynamic nature of these materials, highlighting that PBAs act as precatalytic structures capable of undergoing surface reconstruction under operating conditions, leading to the formation of secondary active phases. This aspect helps reconcile discrepancies reported in the literature and establishes a direct link between synthesis conditions, morphological defects, and electrochemical performance. From a comparative standpoint with the current state of the art, it is evident that progress in PBAs remains limited by the lack of standardization in experimental protocols, the use of simplified electrolytes, and the scarce evaluation under real seawater conditions, including the effect of different cations interacting with the PBA structure. These limitations hinder the identification of clear trends and emphasize the need for more rigorous and representative evaluation frameworks. Their ability to integrate catalysis, ion transport, and electrolyte regulation opens new opportunities for the development of hybrid architectures and high-entropy PBAs. In particular, the rational control of composition, structure, and operando transformations emerges as a key factor for simultaneously optimizing activity, selectivity, and stability under extreme conditions.

Overall, this manuscript positions PBAs as an emerging platform whose true potential does not lie in maximizing a single performance parameter, but in their ability to operate robustly in complex environments, contributing to the development of sustainable electrochemical technologies under realistic conditions.

## Figures and Tables

**Table 1:** Comparative electrochemical performance of PBA-based materials under seawater and chloride-rich electrolysis conditions.

PBA-Based Material	Electrode Role/Reaction	Saline Electrolyte	Medium Classification	Main Electrochemical Performance	Stability	Selectivity/Saline-Specific Behaviour	Ref.
CoHCF (CoFe-PB)	Anode/OER	Seawater	Natural seawater	Catalyzes seawater oxidation; OER and chloride oxidation occur competitively	Not reported	Both water and chloride oxidation were detected, demonstrating insufficient intrinsic OER/CER selectivity	[115]
Ni–Co@Fe–Co PBA	Cathode/HER	1 M KOH + 0.5 M NaCl	Alkaline simulated seawater	η_10_ = 183 mV; full PBA-based electrolyzer: 30 mA cm^−2^ at 1.60 V	HER cathode retained 98.4% after 24 h; full cell showed no obvious attenuation over 100 h	Full PBA-based cell exhibited approximately 100% Faradaic efficiency for H_2_/O_2_ evolution	[16]
CoFePBA/NCNT || Ni/NCT	Anode/Overall seawater splitting	1 M KOH + 0.5 M NaCl	Alkaline simulated seawater	Cell voltage = 1.71 V at 10 mA cm^−2^	50 h	PBA is used at the OER electrode; cathode is Ni/NCT. Performance therefore represents a hybrid rather than an all-PBA electrolyzer	[116]
NiFe-PBA || NiMoN	PBA anode/Overall seawater splitting	Alkaline seawater	Alkaline seawater	Cell voltage = 1.782 V at 500 mA cm^−2^	100 h	Hierarchically hollow NiFe-PBA provides the OER functionality, whereas NiMoN performs HER	[114]
NiHCF-based PBA	Anode/OER	Alkaline seawater	Alkaline seawater	η at 1 A cm^−2^ = 349 mV	Negligible degradation over 1000 h at 1 A cm^−2^	Dynamic structural evolution/self-reconstruction enables sustained ampere-level seawater oxidation	[107]
NiHCF-1/NiHCF-2/CuHCF	Anode/OER vs. CER	Acidified simulated seawater	Acidified simulated seawater	Study focused primarily on reaction selectivity rather than high-current electrolysis performance	Not reported	OER selectivity: 26.47% (NiHCF-1), 14.56% (NiHCF-2), and 10.94% (CuHCF); structural water was identified as a descriptor influencing OER selectivity	[112]
N_2_–NiCoFe-PBA	Anode/OER	Alkaline simulated seawater and alkaline natural seawater	Simulated + natural seawater	η_10_ = 293 mV in simulated seawater and 323 mV in natural seawater; parent NiCoFe-PBA: 405 and 434 mV, respectively	>110 h at 250 mA cm^−2^ in 1 M KOH + 2.0 M NaCl	CN-vacancy engineering promotes reconstruction toward active NiCoFeOOH and improves tolerance to Cl^−^	[92]
Pt/NiFePBA	Cathode/HER	6 M NaOH + saturated NaCl	Hypersaline model electrolyte	Stable HER at −500 mA cm^−2^	Continuous operation demonstrated; exact duration NR in abstract	Released [Fe(CN)_6_]^4−^ modifies NaCl crystallization and suppresses salt deposition on the cathode (“halophobic” behaviour)	[108]
CoFePBA-N	Anode/OER	1 M KOH + 0.5 M NaCl/1 M KOH + natural seawater	Alkaline simulated seawater	η_10_ = 344 mV simulated seawater; 374 mV natural seawater. Tafel = 47.9 mV dec^−1^ in simulated seawater	≈270 h in alkaline natural seawater	High Cl^−^ tolerance; OER kinetics and active-site accessibility are only slightly affected by chloride.	[126]
Ni(OH)_2_/NiFe-PBA	Anode/OER	Alkaline seawater	Alkaline seawater	η_10_ = 292 mV	1000 h at 500 mA cm^−2^	Surface-reconstructed Fe(CN)_6_^3−^ layer repels Cl^−^ and suppresses chloride oxidation.	[127]
NiMoS_x_@NiMnFe-PBA	HER + OER/overall splitting	Alkaline seawater	Alkaline seawater	HER: η_10_ = 71 mV; OER: η_10_ = 260 mV; overall: 1.513 V at 10 mA cm^−2^	2500 h at 500 mA cm^−2^	NiMnFe-PBA shell provides Cl^−^ repulsion and protects the catalytic core from corrosion.	[128]
PBA-350 (FeMn@CoNi-derived PBA	Cathode/HER	1 M KOH + 0.5 M NaCl	Alkaline simulated seawater	η_100_ = 159.5 mV in simulated seawater	Saline stability demonstrated; alkaline test: 100 h at −50 mA cm^−2^	Hydration-layer-mediated Cl^−^ repulsion; near-unity H_2_ FE with no significant competing cathodic reaction.	[129]
R-MnFeO_x_/PBA	Anode/OER	Simulated seawater	Simulated seawater	η_10_ = 201 mV; MEA Faradaic efficiency up to 97.6%	1150 h at 1000 mA cm^−2^	Reconstruction promotes selective OER; FE up to 97.6%; quantitative CER suppression not specifically reported.	[130]
NiFe-PBA/NiFe/CNTs	Anode/OER	Alkaline seawater	Alkaline seawater	η_1000_ = 368 mV in seawater	150 h at 200 mA cm^−2^	Carbon shell mitigates Cl^−^-induced corrosion and preserves OER stability.	[131]
NiFe-PBA-gel-cal (Fe_3_O_4_/NiC_x_)	HER + OER/overall splitting	1 M KOH + 0.5 M NaCl	also tested with alkaline real seawater	Overall: 1.66 V at 100 mA cm^−2^ in simulated seawater; real alkaline seawater ≈ 1.64 V at 100 mA cm^−2^	≈50 h overall; real seawater testing also reported	No detectable Cl_2_ or ClO^−^/HOCl; reconstructed NiOOH_2−x_ shell limits Cl^−^ contact and improves corrosion resistance	[132]
Fe(OH)_3_/MoNiO_x_ || PBA-derived	HER + OER/overall splitting	1 M KOH + seawater	Alkaline seawater	OER η_10_ = 206 mV; HER η_10_ = 337 mV; overall 1.575 V at 10 mA cm^−2^	>120 h at 100 mA cm^−2^	Stable in alkaline seawater; anodic reconstruction to Ni(Fe)OOH. Quantitative OER/CER selectivity was not reported	[133]

## Data Availability

No new data were created or analyzed in this study. Data sharing is not applicable to this article.
